# Joined-up governance for more complementary interactions between expanding artisanal small-scale gold mining and agriculture: Insights from Ghana

**DOI:** 10.1371/journal.pone.0298392

**Published:** 2024-04-04

**Authors:** Enoch Adranyi, Lindsay C. Stringer, Henrice Altink

**Affiliations:** 1 Department of Politics, and Department of Environment and Geography, Interdisciplinary Global Development Centre (IGDC), University of York, York, United Kingdom; 2 Department of Environment and Geography, York Environmental Sustainability Institute, University of York, York, United Kingdom; 3 Department of History, Interdisciplinary Global Development Centre (IGDC), University of York, York, United Kingdom; BOKU: Universitat fur Bodenkultur Wien, AUSTRIA

## Abstract

Rising gold prices have led artisanal and small-scale gold mining (ASGM) operations to proliferate in sub-Saharan Africa, extending into agricultural areas. Little is known about the interactions between agriculture and mining in these new frontiers. This study aimed to investigate the impacts of ASGM on natural and physical livelihood capitals, ASGM’s interactions with agriculture at household, community and institutional levels and the drivers underpinning those interactions, and the policy implications for the co-existence of sustainable agriculture and ASGM. Alongside literature review, field-work took place in Atiwa West District and Koforidua, Ghana using environmental field surveys, questionnaires, focus group discussions and interviews. Questionnaire and field survey data were analysed using descriptive statistics, with thematic analysis of interviews and focus group data. Findings revealed that most miners were unregulated, mined irresponsibly and degraded land, waterways, and farm roads. Over one-third of farmers (38%) suffered land degradation, and 79% of affected farmers’ lands were not reclaimed. Farmers diversified into ASGM, and mining proceeds boosted farming. Young farmers (18–40 years) shifted into ASGM full-time because it is more lucrative. Yet, ASGM is not replacing agriculture: cocoa farming remains a vital economic activity. Informal ASGM generates short-term income at household level for some but imposes long-term costs at community level, linked to cumulative loss of agricultural land and degradation of forest areas and water bodies, creating tensions, and increasing vulnerability. Financial hardships faced by farmers, landowners’ desire to benefit directly from gold and lack of law enforcement drive informal ASGM. There are no institutional linkages between the agricultural and mining sectors. More joined up governance across agriculture and mining is needed and between formal and informal (traditional) institutions. ASGM should be incorporated into broader rural development policy reforms that support farmers, incentivise miners to operate legally and responsibly and ensure effective stakeholder engagement.

## 1. Introduction

Artisanal and Small-scale Mining (ASM)–a low-tech, labour intensive, low investment mineral extraction and processing activity—has grown to become a viable livelihood of global significance in recent decades [1]. A livelihood comprises the capabilities, material and social assets and activities required to make a living [2]. An estimated 40 million people work in ASM across 80 countries in the global south and about 10 million ASM operators exist in 40 countries in Africa [3]. ASM is focused on various precious minerals and stones, and in particular gold. Artisanal and small-scale gold mining (ASGM) represents an estimated 20% of annual global production [3]. The upsurge in ASGM since the early 2000s is due to steady rises in gold prices, coupled with limited rural employment avenues [4]. Sub-Saharan Africa (SSA) is a leading region in ASGM operations, where the growing industry provides substantial economic benefits [5]. Ghana is Africa’s largest gold producer and the sixth largest in the world, producing 138.7 tonnes in 2021 [6]. In Ghana, ASGM accounts for c. 35% of total annual gold production [4, p.3] a significant increase from the c. 5% (<0.62 tonnes) in the 1990s.

ASGM targets ore deposits that are often close to the land surface and can be extracted easily and quickly. Miners are thus highly mobile in chase for new discoveries or higher-grade ore deposits, which can result in rapid shifts in local population and emergence of new settlements over short periods [7]. ASGM’s rise raises important questions worldwide about how ASGM interacts with and impacts pre-existing agricultural livelihoods. ASGM can negatively affect agriculture through deforestation, water pollution, farmland degradation, input competition and a shift of farm labour/resources into mining and can thereby increase chances of conflict and impact the health of residents [8]. ASGM can also positively complement agriculture. Agricultural income can provide capital to enter into ASGM, and ASGM income can be used to purchase farm inputs, hire labour, and maintain farms, helping to supplement farm incomes and providing the possibility that ASGM emergence can help accelerate agricultural development [9]. Malone et al. 2021 examined the coexistence between ASGM and farming and fishing in Peru. They found that ASGM could coexist with farming but the practicality of this over the long term was threatened by the rapid pace of ASGM growth, immigration and social change, alongside uncertainty about water contamination that could undermine other livelihoods [10]. In Guyana, illegal miners took over territory and pushed out agriculture, undermining traditional livelihoods and forcing locals to shift into mining [11, 12].

Agriculture in SSA is exposed to multiple stresses and shocks [13, 14]. Mismanagement and poor land use practices are common, and lead to land degradation. Here, land incorporates soils, vegetation, and water [15, 16] with land degradation defined as ‘‘a negative trend in land condition, caused by direct or indirect human-induced processes including anthropogenic climate change, expressed as long-term reduction or loss of at least one of the following: biological productivity, ecological integrity, or value to humans” (Shukla et al., 2019, p4), [17]. ASGM poses an extra burden on agriculture because mined lands can render previously fertile lands unsuitable for cropping [18]. Only effective reclamation (that ensures physical stability, waste management, and acceptable land uses) can return mined areas to an acceptable condition for continued productive use [19, 20]. Hilson and Garforth (2012) found in Mali and Ghana that diminishing returns from agriculture provided the overarching incentive for farm families to branch out into ASGM. Others simply enter ASGM because of their belief they will earn more revenues and achieve their dreams from doing so [21, 22].

Existing studies on ASGM and agriculture interactions in SSA include case studies from Tanzania [23] Zimbabwe [24] Ghana [25] and Mozambique [26] which showcase negative environmental effects of ASGM on neighbouring farms. Studies have also noted the ensuing competition between ASGM and agriculture in the acquisition and use of land, capital, labour, and water, resulting in resource-use conflicts [22, 27]. ASGM can also generate positive relationship with agriculture as noted in case studies from Sierra Leone [28] Malawi [29] Mali and Ghana [30] Zimbabwe [31]. These studies found that smallholder farmers often shift between farming and mining and use small-scale mining income to support agriculture and enhance productivity. Such complementarities are important for poor smallholder farmers for whom poor access to credit and markets often affect agricultural productivity. A few studies have also examined both the positive and negative dynamics between the small-scale mining and agriculture [22, 27].

Scholarship on ASGM-agriculture relationship, however, has largely focussed on describing negative or positive relations as observed in field studies, providing snapshot views of happenings on the ground [30]. Studies have not explored for instance if positive relations can continue over the long term; nor have they sought to understand how dynamics in each sector may affect the other. Furthermore, existing literature has also not paid attention to the institutional linkages between ASGM and agriculture [21, 22]. Understanding of these relationships and their dynamics is needed to foster a more mutually supportive relationship between ASGM and agriculture. This study addresses these gaps by exploring agriculture-ASGM interactions at household, community, and institutional levels, to find out whether households and communities in cocoa farming areas in the new frontiers of ASGM can embrace both mining and agriculture to advance their livelihoods, not just in the short–but also long–term. It examines the socio-economic, biophysical, customary, institutional, and political factors that shape relations between ASGM and agriculture in cocoa farming areas. In doing so, this paper offers a more nuanced understanding of the dynamics of ASGM-agriculture interactions in new mining frontier regions.

ASGM’s rise in Ghana in the past two decades is characterised by operations on land, on riverbanks and in riverbeds by increasing number of both local miners and foreign illegal miners using sophisticated machinery [32]. Governance framework for ASGM in Ghana—the Minerals and Mining Act, 2006 (Act 703) and the Minerals Commission Act, 1993 (Act 450)—stipulates that all mineral deposits in Ghana belong to the state, which via the Minerals Commission (MC), has sole responsibility to grant mineral rights, controlling mineral access and concession licensing [33]. Only citizens of 18 years and above can obtain small-scale mining licenses. However, miners’ access to mineral rich lands is highly influenced by Ghana’s complex land ownership system. There are government lands, customary lands, and private lands [34]. Customary lands constitute c.80% of Ghana’s land surface and are administered by family/clan heads and chiefs [35]. This distinction–between mineral rights and land rights–poses challenges to mining administration [36]. Many customary landowners who think they ought to benefit directly from the minerals are able to directly deal with ASGM miners, granting miners access to lands to mine outside of the direct control of the state. Transactions are quick and informal, making it popular with community members and illegal ASGM miners alike [37, 38].

ASGM in Ghana is rapidly extending into areas traditionally known for agriculture such as cocoa production. This is the case in our study area–Atiwa West district, raising questions about the impact of ASGM on cocoa farming and agriculture more generally [39]. Agriculture is the main source of livelihood in Ghana’s rural communities [22]. It accounted for 19.7% of GDP, US$ 77.59 billion, in 2021 [40, 41] and is the largest employer, with ~77% of rural dwellers in Ghana making a living through agriculture [42]. Cocoa is the country’s most important agricultural export delivering an average of 30% of the country’s total export earnings [43]. Yet, Ghana remains a country in food deficit [27]. Smallholders face numerous constraints that significantly affect agricultural productivity and keep farmers poor e.g., lack of farm inputs [44, 45]. The shifting nature of ASGM activities makes the relation between ASGM and agriculture complex, and its dynamics remains incompletely explored and understood.

Considering that both agriculture and ASGM are important sources of livelihoods for many rural people, there is a need for greater understanding of the ASGM–agriculture relationship to ensure their balanced interaction, producing social and economic development without disrupting rural livelihoods. Based on the problems described above, the overarching aim of this paper is to investigate ASGM’s impact on natural and physical livelihood capitals and interactions with agriculture, in order to understand policy implications for a positive co-existence of sustainable agriculture and ASGM. To achieve this, the following three research questions have been established: i) How have agricultural lands, water resources and local infrastructure been altered through ASGM activities and what agriculture-ASGM interactions occur at the household, community, and institutional levels? ii) What individual and societal drivers have shaped the interactions between ASGM and agriculture and brought about changes in the natural and physical livelihood capitals? iii) What are the policy implications of the agriculture-ASGM interactions?

## 2. Methodology

### 2.1. Study area description

**Fig 1:**
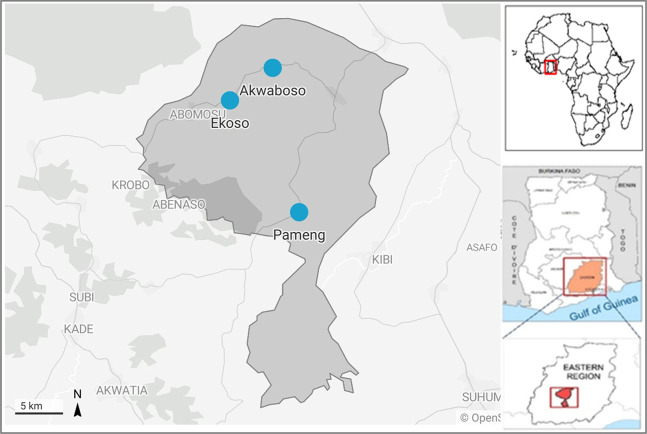
Map of Atiwa West district, showing the selected communities. Source: Authors’ construct with Datawrapper.

Ghana has 31 million inhabitants and is made up of 16 administrative regions. Atiwa West District in the Eastern Region in southern Ghana (Fig 1) was selected as the case study area given its recent growth in ASGM activity. The district is about 118 km north of Accra, Ghana’s capital city. Following commercial discovery of huge gold deposits (7.4 million oz) in the neighbouring Birim North District by Newmont Mining Inc. in 2010, [46] ASGM miners (both legal and illegal) have since been exploring and mining gold in Atiwa West. In Ghana, illegal miners are those who mine without a valid license and have no authorised concessions of their own and their activities are called *galamsey* (gather them and sell) in local parlance. Legal miners are those who have a small-scale mining license and operate in accordance with mining regulations and within approved concessions of no more than 25 acres, as stipulated in Act 703 [47, 48].

Due to its abundant fertile lands and optimum local climatic conditions, regulated by the lush Atewa Range Forest, the district’s 660.7 km^2^ area is known for agriculture and forms part of the enclave producing the region’s highest annual yield of cocoa (*Theobroma cacao*) [45, 49]. Media reports indicate that ASGM is expanding rapidly in Atiwa West district, with activities affecting farms and the broader environment, putting not just food and cocoa farms at risk but also destroying forest areas that provide important ecosystem services [39, 50]. Nevertheless, independent academic analysis has not yet corroborated this. Given Ghana’s contribution to the global growth of ASGM activities, this case study has broader relevance and can inform the development of policies that foster more sustainable agriculture-ASGM interactions.

### 2.2. Methods

To answer the research questions in the present study, an explanatory research design and mixed qualitative-quantitative research approaches were applied in collecting, integrating, and analyzing empirical data, to gain deeper understanding to the research problem. To answer the first research question, a combination of environmental field survey, household questionnaire survey, focus group discussions (FGDs) and interviews were applied to collect data. The second research question was assessed using focus group discussion, interviews, and household surveys. These methods ensured consultative and participatory approaches of engagement with individual households, community groups and institutions. The research question three was addressed by discussing findings from research questions one and two within the context of Ghana’s legal and policy framework on ASGM. This helped to explore policy options for complementary ASGM-Agriculture interactions along household, community, and institutional levels. Table 1 highlights the different sampling techniques, methods, tools and data analyses used to address the research questions.

**Table 1:** Research methods and sampling techniques applied.

Research Question	Method	Sampling method	Sampling size	Data capture tools	Data analysis
1, 2	Face-to-face household survey with semi-structured questionnaire	Simple random sampling	360* (117-Akwabuoso; 121-Ekorso; 122-Pameng) c.30 mins each	Questionnaire; Qualtrics Offline Surveys; Tablets; Field notebook	Descriptive statistics, using SPSS v27^**+**^
1, 2	Focus group discussions (FGDs) with farmers	Purposive sampling	1 FGD per community; 10 members each (7 men, 3 women); 1 hour each	Voice recorder; Smartphone camera; FGD schedule	Thematic analysis (by manual coding)
1	Environmental field survey in communities and mined farms—visits with farmers	Transect walk; field observation; Pace counts; visual estimation of length and breadth of site	Within each community ^**+**^ 3 farms per community	Voice recorder; smartphone camera; environmental checklist	Descriptive statistics using Excel**
2	Interviews with ASGM miners	Purposive sampling	5 per community (3 men 2 women); 30 mins each	Voice recorder; Interview schedule	Data translated & transcribed; Thematic analysis
2	Stakeholder interviews	Purposive sampling	11 in total; 30 mins each	Voice recorder Interview schedule	Data translated & transcribed; Thematic analysis
3	Document review	Results from questions 1 & 2 purposively compared with laws/policies on ASGM in Ghana	-	-	Results interpreted at multiple scale within the context of existing laws/policies on ASGM in Ghana

* Information from District agricultural officers indicated ASGM was present in several communities, so the study assumed 1/3 of the district population was experiencing ASGM. The district population is 61,219, so 1/3^rd^ is = 20,406 (GSS 2021) [51]. Number of individuals per household is approximately 4 (GSS 2021, p. 83), so our sample population was 5,102 households (20406/4). The sample size thus was determined at 360 based on a statistical confidence level of 95%; margin of error of 5% as well as considering time and cost (Bryman and Bell, 2007; Qualtrics.com, 2022) [52, 53]. Estimate of community population obtained through discussion with district agricultural officers: Akwabuoso–~3,000; Ekorso–~3,000; and Pameng–~4,000.

+ Statistical Package for Social Sciences software (SPSS) version 27.

**Microsoft Excel Version 2207, released in 2022.

Fieldwork was undertaken between October and December 2021 in Akwabouso, Ekorso and Pameng. These communities were selected because they are all farming communities experiencing ASGM operations; are accessible by road; residents were willing to participate in the study; and between them they cover the district’s three Area Councils (Kwabeng, Abomosu and Akropong) ensuring data was gathered across the district. The three communities were chosen following a review of online news publications [39, 50, 54, 55] use of Google Earth imagery to observe recent mining activities, and communications with a district agricultural extension officer. The stakeholder interviews were conducted in the communities but also in Kwabeng–the Atiwa West district capital, and Koforidua, the Eastern regional capital.

Questionnaires were conducted with residents of the three communities to gather data on the everyday reality of farming and gold mining in the district. After the questionnaires, Focus Group Discussions (FGDs) were used for clarification, validation of findings and to provide forward-looking insights. Farmers were selected based on their experiences and interest to participate, ensuring representation across genders and age groups. Additionally, environmental field observations were conducted in the communities to identify the prevailing situation of gold mining with a particular focus on the impacts of ASGM on land, waterbodies, and local infrastructure. Some FGD participants whose lands were mined were later visited to conduct environmental field surveys. Afterwards, miners were interviewed. Interviews with miners and FGDs with farmers were conducted to uncover a wide variety of political, socioeconomic, cultural, and environmental factors related to ASGM and its interactions with agriculture. Stakeholder interviews were also conducted with 11 officials (the community and district level interviews included: two district agricultural officers; one Atiwa West District Assembly official, one cocoa purchasing officer/traditional leader; one educationist; one chairman of a farmers’ cooperative/traditional leader; while the regional level interviews at Koforidua included two officers from the Minerals Commission, one from the Environmental Protection Agency (EPA), one from the Ministry of Food and Agriculture, and one from the Lands Commission). Here, participants were identified based on their knowledge and experience. This expert judgment process was used to verify the reliability of the information obtained in the interviews with the miners and farmers and to explore the nature of institutional linkages and challenges. Interviews continued until we reached saturation, with no new information being received [56].

Regarding data analysis, quantitative data collected in questionnaire surveys using Qualtrics were transferred to Scientific Package for Social Sciences (SPSS) version 27 and analysed using descriptive statistics. Quantitative data from environmental field surveys were analysed using descriptive statistics in MS Excel, version 2207. Findings are presented in tables and in the text. Analysed data from questionnaire surveys are also in S1 Appendix. Qualitative data (interviews and FGDs recordings) were, where required, translated from Twi into English and transcribed (as data collection in the communities was in Twi). Thematic analysis was then applied, where data were manually coded and indexed and major themes identified from recurring ideas were described, including extraction of illustrative quotations. Appropriate quotes from farmers, miners and experts were used to provide more emphasis to enrich the discussions. In line with ethics approvals, participants are anonymous but titles of those interviewed in their official capacities are provided. All quotes in Twi were translated by the first author. Primary results were triangulated with data from published documents, newspaper articles and laws and policies that were reviewed. Policy implication deductions were made from the findings.

This study was approved by the Ethics Committee of the authors’ University. The ethics process ensured that participants were well informed about the study, their confidentiality and anonymity was assured, and participants’ consent was secured (verbally or through signing a consent form) prior to the commencement of the data collection. Participants were also told that their data would be carefully stored and who would have access to it. Participants were further made aware that involvement in the research was without compensations, and that they could withdraw at any stage up to the point of data analysis.

Field-work was restricted to one administrative region, one district and three communities within the district due to limited time and funding. The study did not include laboratory testing of samples to detect chemical contamination of soil, water, plants, and humans caused by ASGM, since the focus was on the governance and policy implications of the interactions, rather than on technical environmental impact assessments.

## 3. Results and discussion

### 3.1. Alterations to agricultural land, water resources, and Infrastructure and ASGM-agriculture interactions at household, community and institutional levels

In this section, ASGM interactions with agriculture are analysed at household, community, and institutional levels. Each level of interaction is examined in relation to ASGM’s impacts on agricultural land, water resources and infrastructure.

#### 3.1.1. ASGM-Agriculture interaction at household level

Traditional small-scale food and cash crop farming dominates land use and 80% of farmers farm cocoa (S1 Appendix). Farmers also cultivate oil palm, tubers, rice, plantain, and vegetables for subsistence and for trade. Farmers cultivate under different land tenure arrangements (Table 2) while average landholdings cover 2.74 ha (6.76 acres), similar to elsewhere in Africa [21].

**Table 2:** Kinds of land tenure arrangements held over farmland based on data from 259 respondents.

Types of land tenure	Percentage (%)
Owner (inherited)	49
Owner (gifted)	8
Owner (private)	6
Rented	1
Share cropping	34
Caretaker	2
Total	100

**Fig 2:**
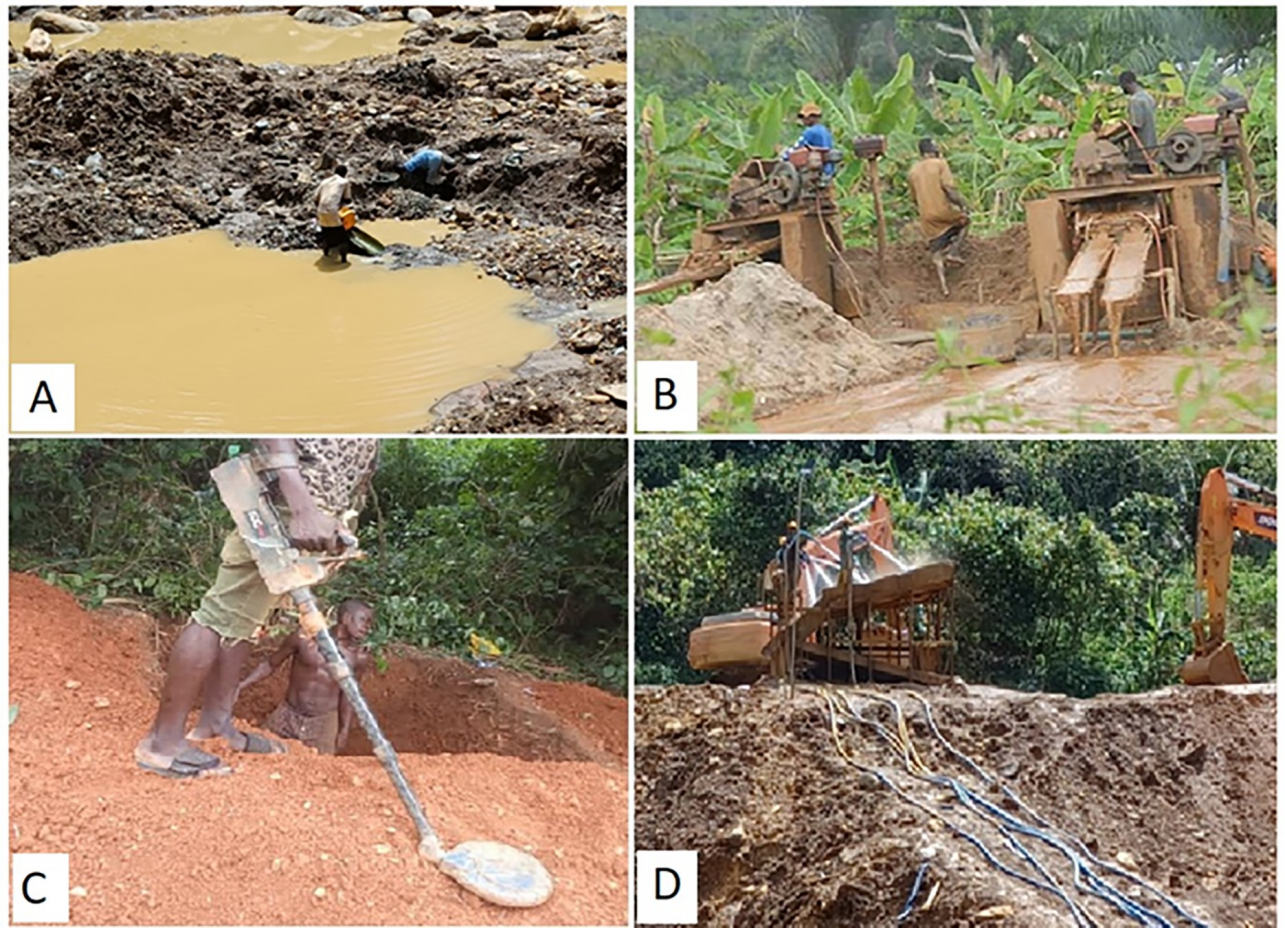
Types of ASGM methods practised in the study area. A–Mine waste scavenging (Kuli-kuli), Pameng–picture by first author. B–Washing board with water pump (Suagum), Ekorso–picture by research assistant Jeffrey. C–Metal detector mining (kwee-kwee), Pameng–picture by research assistant Sylvester. D–Excavator, loader, and sluice (Washing plant), Pameng–picture by first author.

Interviews and field surveys showed that ASGM in Atiwa West is intensive and marked by use of sophisticated machinery. Most ASGM operations are informal, without licenses and permits. With the influx of ASGM entrepreneurs into the communities, residents engaged in ASGM using diverse approaches (Table 3) and practised surface mining using various operational methods across different locations within the landscape (Fig 2; Table 4). In return, farmers generated significant income to support their farming activities, thus permitting movement of labour and finance between the two sectors. Questionnaires showed 31% of farmers branched out into ASGM (farmers, n = 219; farmers diversified into ASGM, n = 55) (also see S1 Appendix). Similar linkages have been found elsewhere [48, 57]. Questionnaires showed miners mostly leased lands from landowners (73%), enabling landlord farmers to raise considerable funds to support their livelihoods. Land invasion occurs on minor scale (27%).

**Table 3:** ASGM practice approaches.

ASGM operational type	Percentage, n = 118
ASGM only on permanent basis	31.4
ASGM mainly with some farming elsewhere or shifts between farming and ASGM seasonally	46.6
Just as investor (do not physically take part)	1.7
Other (does non-farm job with mining as a support)	20.3

**Table 4:** ASGM methods practised in the study area, based on FGDs, interviews and field surveys.

Method and machinery used	Landscape location	Local name
Excavator/pickaxe & shovel, pan, washing board, water pump. Soil is dug and heaped, gangs hired to fetch and wash soil.	Farmlands near waterways, low lying areas and valleys containing alluvial gold deposits.	*Suagum*
Locally–made floating machines with suction tubes–dredgers–that draw gold bearing mud from riverbeds	On or along rivers/other waterbodies.	*Chanfan*
Excavator, loader, sluice, trommel, water pumps/engine. Excavator heaps soil, loader collects soil into wash plant (machine intensive and less labour).	Any farmland that bears gold, also targets alluvial deposits.	*Washing plant*
Use pickaxe and shovel to collect mud and wash with pan mostly or washing board.	Farmland near waterways, low lying areas, valleys.	*Foo-foo/dig and wash*
Use pickaxe, shovel, and pans to scavenge mine waste for residual gold.	Active or derelict small-scale mining sites.	*Kuli-kuli*
Metal detectors, pickaxe, shovel, and pans are used to expose and loosen the ground to enable detectors to find and collect the nuggets	All types of farmlands—cocoa farms, fallow fields; within communities.	*Kwee-kwee*

Miners applied trial and error techniques, based on their field know-how, to undertake mining which resulted in hit and miss outcomes and mounted environmental costs. Questionnaires and interviews showed most miners (80%) are 18–40 years old and ASGM is strongly gendered: men mostly engage in digging and women tend to carry the dug materials to washing centres. Ore-containing soil extracted after washing is mixed with mercury to form a mercury-gold amalgam, then burned to vapourise the mercury, leaving behind a semi-pure form of gold. Some miners use cyanide to extract gold. These chemicals, once released into the environment, can contaminate soils, waterways, and the atmosphere.

*Agricultural land*. Farmlands constitute important natural capital in local livelihoods. Yet informal ASGM has affected farmlands. Household questionnaire surveys revealed that 38% of respondents deemed their farmlands were degraded by ASGM. Affected farmers lost on average one-third (32%) of their holdings to ASGM degradation (Table 5). Of the affected farmers, 79% reported unreclaimed mined areas, while in many cases where reclamation was attempted, it was not done properly. Surfaces were left covered in gravel and other subterranean materials instead of topsoil, rendering land unfit for farming.

**Table 5:** Proportion of farmers affected by ASGM, and the extent and forms of land degradation occurring.

	Farmland degraded by ASGM (n = 220). NO YES	Percentage of household farmland degraded (mean land area degraded, n = 80; mean of total farmland, n = 219, in hectares)	Dominant forms of land degradation
**Aggregate score**	62% 38%	32% (0.87; 2.74)	
**Akwabuoso**	63% 37%	30% (0.67; 2.25)	***Soil degradation***: Degraded lands covered in shrubs, heaps of mine waste, rainfed pits with some containing dangerous water-loving animals, overflow of wastewater into cocoa farms ***Vegetation degradation***: Clearance of cocoa farms and deforestation of fallow fields. ***Water resources degradation***: river/stream pollution with mine waste.
**Ekorso**	68% 33%	25% (0.93; 3.73)	***Soil degradation***: Denuded landscape, bare ground covered in gravel, mine waste and huge pits/ponds, soil exposed to wind and water erosion, depleted topsoil/organic matter, holes dug in cocoa farms, likely chemical pollution from mercury/cyanide use. ***Vegetation degradation***: Deforestation/clearance of virgin farmlands, Clearance of cocoa farms. ***Water resources degradation***: river/stream pollution with mine waste.
**Pameng**	55% 45%	47% (1.04; 2.23)	***Soil degradation***: Bare ground experiencing water erosion, mine heaps, mine holes dug within community, in between cocoa crops causing plants to wilt, likely chemical pollution from mercury/cyanide use. ***Vegetation degradation***: Deforestation, clearance of cocoa farms, fallow fields, and marshy areas. ***Water resources degradation***: pollution of streams.

*Water resources*. Water resources are also vital natural capital that miners depend on as much as farmers. Miners consider rivers, riverbanks, low-lying areas and valleys as sources of alluvial gold so have mined those areas, with many water resources becoming heavily polluted, despite regulation stipulating that a 300 m buffer zone next to water bodies should not be mined [58]. Miners use the polluted water to wash the soil, but they purchased drinking water. Farmers reported that they no longer fetch water from these rivers/streams to drink but instead buy sachet water and are unable to use polluted sources to irrigate vegetable farms during dry seasons. Those who previously used polluted water on their crops reported that plants wilted, possibly due to excessive mud, chemicals, and fuel waste contamination.

*Finance and infrastructure*. Questionnaire surveys and FGDs revealed that at the household level, informal ASGM enabled customary landowners and households that ventured into mining to attain financial and physical livelihood assets to improve their livelihoods (Table 6). Interviews with artisanal miners showed 1 carat of gold is sold for GHS 60 [i.e. 1 g of gold = US$60; 5 carats = 1 gram; 5GHS = 1US$].

**Table 6:** Assets households acquired as a result of significant contribution from ASGM income (more than 50% of the cost was covered by ASGM income). Data based on questionnaires.

Assets acquired	Frequency
TV	40
Radio	32
Mobile phone	55
Shop	18
Car/truck/van	9
Motorbike/motor tricycle	20
Bicycle	2
Motor/manual boat	0
House	28
Land for building	32
Farmland	5

#### 3.1.2. ASGM-Agriculture interactions at community level

**Fig 3:**
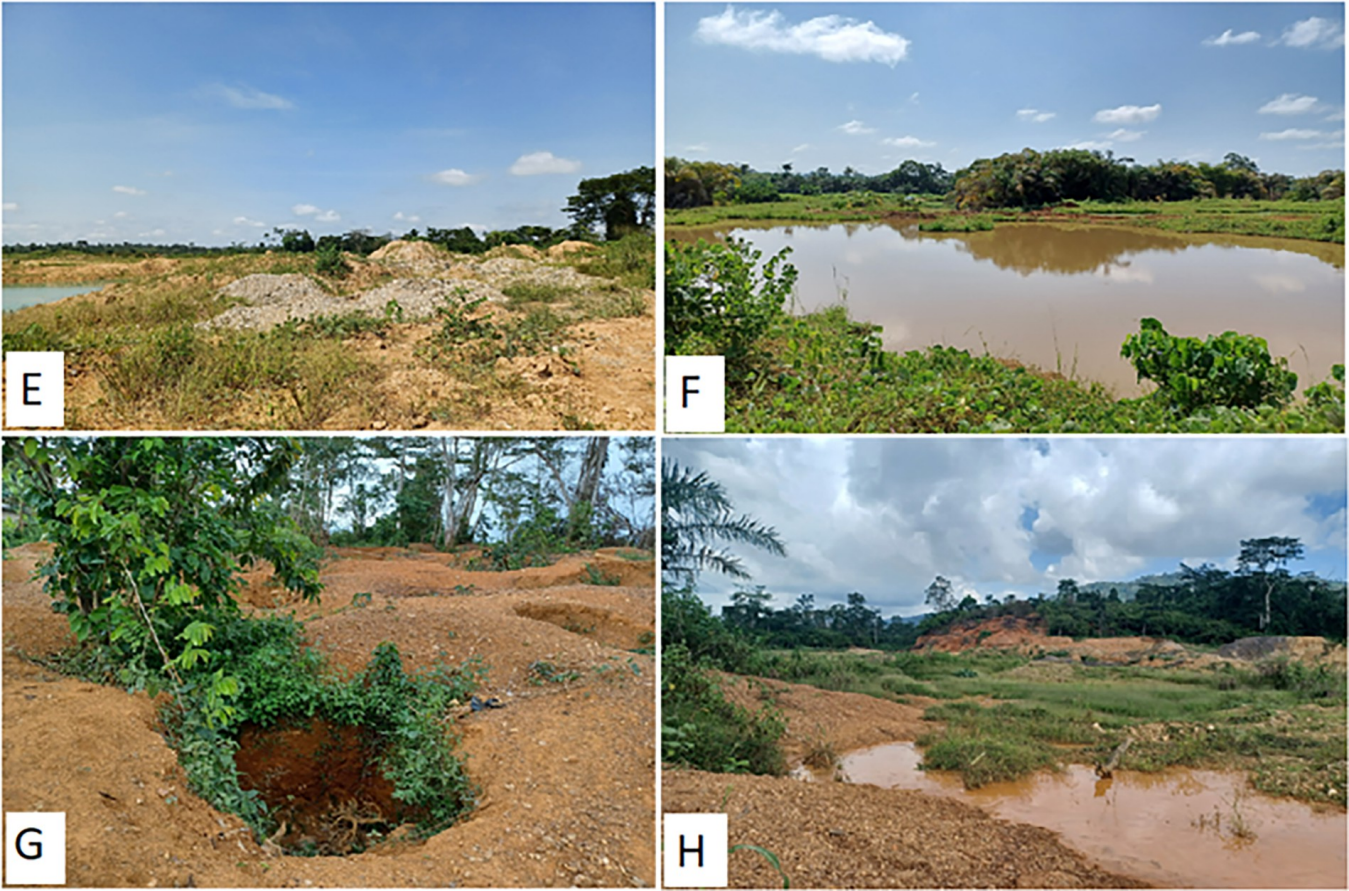
Degraded farmlands in the study area. E–Mine heaps, pond and weeds at an abandoned mine site, Ekorso. F–Mine pit turned into rainfed pond stretching over 40m in diameter, Ekorso. G–A farmland mined using metal detectors, Pameng. H–A low lying area mined using excavators, Pameng. Source–all pictures taken by the first author.

*Agricultural land*. Field surveys across the communities showed that miners generally did not reclaim lands after mining. Some licensed concession holders did not reclaim land, despite government requirements contained in licensing processes. A given area mined using excavators covered anywhere between an estimated 4 ha and c.20 ha. Field surveys again revealed that ASGM-degraded farmlands were characterised by vegetation and topsoil removal and crop destruction (Table 5), with deformed bare ground and surfaces filled with subterranean rocks and gravel. Heaps of mine waste of various volumes were also present, interspersed with polluted streams, patches of eroded land and pits. Field surveys and interviews with small-scale miners showed that pits created were 4 to c. 40 m in diameter, mostly shallow (<30 m), with many turned into rain-fed ponds (Fig 3).

FGDs revealed that ponds provide habitat for crocodiles, pythons, and other water-loving animals dangerous to livestock and humans, while people have fallen into ponds and died. Ponds have also become breeding grounds for mosquitoes, increasing malaria risk. Field visits revealed overflow of polluted water from mine sites into neighbouring farmlands, affecting crops, while ASGM had also drained groundwater from neighbouring farmlands into mine sites, causing crops to wither. These practices forced neighbouring farmers to lease their farms to the miners to avoid financial loss. These activities, along with the abandonment of miners’ storage structures reduced farmland.

Field surveys showed that miners who use metal detectors operate everywhere including backyards and dig holes of 1–2 m diameter and 1–3 m depth, often leaving them uncovered, with holes dangerous to residents and causing crop withering due to root exposure and reduced access to and availability of groundwater (Fig 3). Farmers noted that metal detector miners come from outside the communities, but mine because they have support from local people. However, there was lack of community level effort to ensure they reclaim lands. Miners are also willing to invade and work during the night. Because miners operate in gangs, farmers are left to deal with them, resulting in tensions, as reported by a 58-year-old male cocoa farmer in a FGD at Ekorso:

‘‘*gold mining guys have destroyed our cocoa farms…our farm roads have been badly damaged*. *As for kwee kwee* [metal detectors], *the operators are outsiders; they invade our cocoa farms to mine*. *You will only discover their activities when you visit your farm; and if you try to confront*, *they can kill you*. *In that sense did you profit or lose*? *So*, *in Atiwa West the gold mining is really affecting the farmers*.”

At community level, FGDs revealed residents are worried, and many women are scared to use routes and farm paths marked by these pits and ponds, while overall, pits, ponds and heaps of sand and the inaccessibility they cause, result in considerable losses of cultivable land area. Older farmers and women who traditionally farm near their communities have been impacted greatly as most mining activities have been carried out on the community fringes. ASGM’s emergence, characterised largely by informal and irresponsible mining practices, caused cumulative degradation and abandonment of farmlands, forest areas and water bodies at the community level.

Yet, in some instances where mining sites were properly reclaimed, FGD participants argued that those areas were suitable for farming and could even enhance yields of tubers. Questionnaires showed 54% of respondents asserting this, probably because mining and reclamation loosen the soil, making it suitable for crops [59]. Usually farmers restart with mixed-crop farming on reclaimed land, introducing cocoa as the fertility improves, as cocoa plants do not thrive on improperly or recently reclaimed sites. Oil palm (*Elaeis guineensis*) was also reported to do well on reclaimed lands, including poorly reclaimed areas [60].

According to the responses of community members to our questionnaire, there was a decrease in farm labour and increased cost of hiring farm labourers due to the emergence of ASGM (Table 7). Most labourers are shifting into ASGM because of its perceived higher profit and because of farmland degradation.

**Table 7:** Residents’ perception of ASGM effects on access to and cost of hiring farm labourers.

	Access to labourers (%), n = 215	Cost of hiring labourers (%), n = 216
Increased	8.4	78.7
Decreased	70.2	2.3
No change	12.6	9.3
Not sure	8.8	9.7

**Fig 4:**
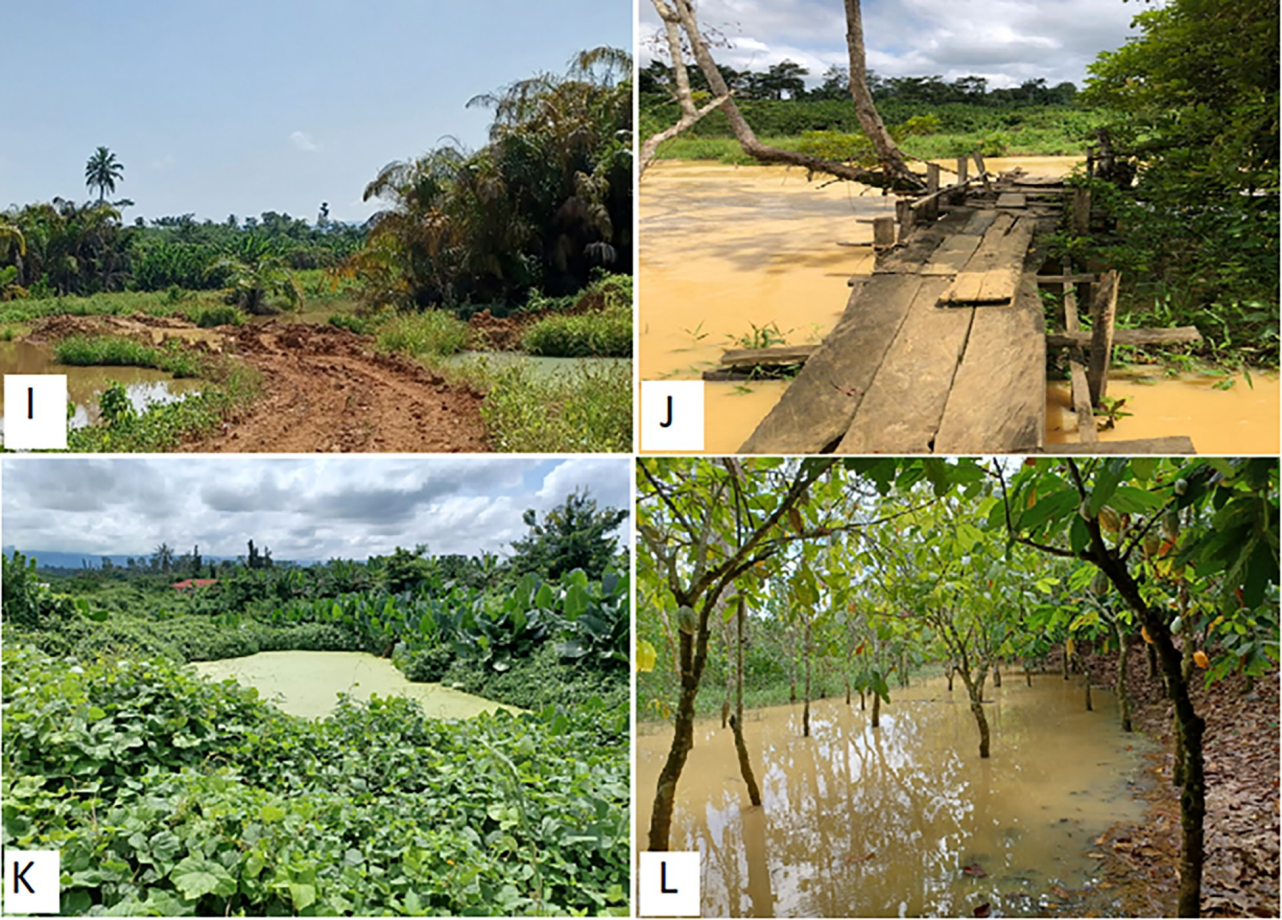
Degradation of natural and physical capitals in the study area. I—Degraded farm road at Ekorso. J—River Birim polluted and farm bridge damaged at Akwabuoso. K—Rainfed pond covered in weeds and algae in a mine site abandoned for > 5 years, Akwabuoso. L—Cocoa farm polluted with mining wastewater from neighbouring farmland at Akwabuoso. Source–all pictures taken by the first author.

*Water resources*. Residents revealed in FGDs that degradation of forests around waterbodies and disruption of river courses by miners increases flood risks to farms. Field surveys showed that in Akwabuoso and Ekorso, the Birim river has turned brown and muddy, with farmers noting a reduction in flow. The sides of the river and parts of the riverbed have been mined, exposing adjoining land and nearby farms to the risk of flooding (Fig 4). Local streams in Ekorso and in Pameng have also been heavily polluted and turned brown by liquid waste discharge from mining sites.

Farmers now must travel further to access alternative water sources for on-farm use or stop vegetable farming. These changes increased farmers’ expenditure, affecting profit margins. A 65-year-old retired male teacher and farmer interviewed in Akwabuoso shared that:

‘‘*…mining has polluted our waters*. *Due to greed*, *miners apply all kinds of inappropriate methods*. *They would divert a river course to let the water pass*, *and mine the riverbed*. *Some would later reconnect the diverted route to the original course*. *Some don’t care and move on*. *Before mining*, *river Birim and Akuku were clear with fish presence*, *and we easily drunk them when returning from the farm*.*”*

Loss of farmlands and waterways can threaten farmers’ livelihoods, as well as creating food security challenges. These communities could become ghost towns at the cessation of mining activities if farmland losses continue unabated. Continued loss of cocoa farms due to increasing ASGM could significantly affect Ghana’s annual production and supply of cocoa to the international market, a concern shared by the leadership of the Ghana Cocoa Board (COCOBOD) [50]. The degradation of waterways and forest areas leads to loss of grounds for fishing, hunting, collection of fuelwood and other non-timber forest products. These changes can increase rural-to-urban migration and exacerbate the poverty of those staying behind.

*Infrastructure*. Field surveys showed local infrastructure/physical capitals damaged by ASGM, including farm roads (in, Akwabuoso, Ekorso and Pameng), and farm bridges (in Akwabuoso), which miners did not repair (Fig 4). This had community level impacts and restricted movement and access to farms. Farmers said miners did not pay any compensation for the harm caused and they were unable to cart enough produce from farms to their homes, causing produce to spoil and available food in the communities to decline.

**Fig 5:**
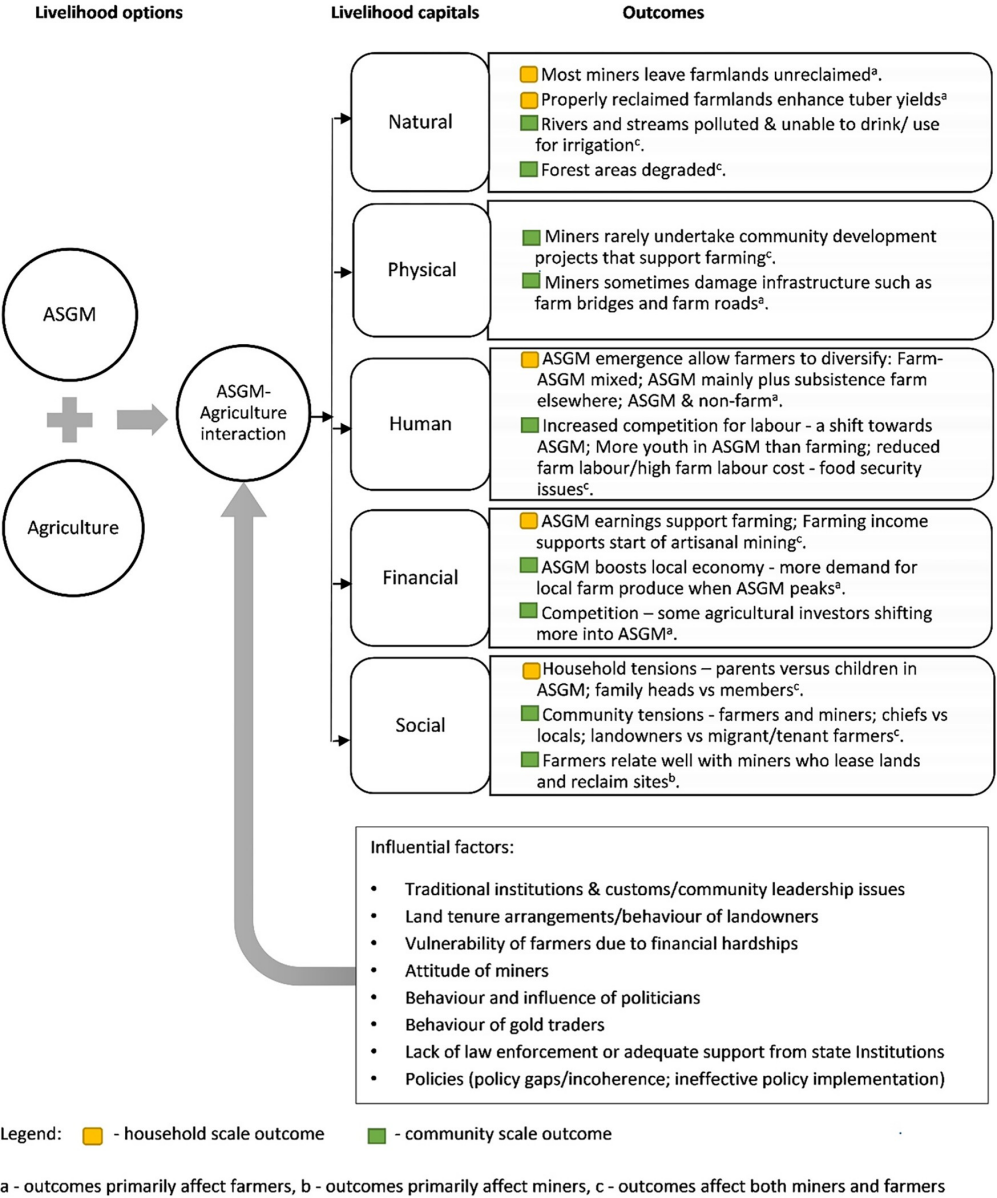
A summary of the ASGM-agriculture interactions across the five livelihood capitals at household and community scales. Source: Authors’ construct.

The interactions between agriculture and ASGM at household and community levels, analysed across the five livelihood capitals–Natural, Physical, Human, Finance and Social are summarized in Fig 5., adapting Scoones’ checklist on the sustainable livelihood framework (Scoones, 1998, p4) [61].

#### 3.1.3. Interaction between agriculture and ASGM at institutional level

Our findings from stakeholder interviews revealed a lack of formal institutional linkages between mining and agriculture at district, regional and national levels. Minerals Commission (MC) officials mentioned they did not have a direct working relationship with the agricultural department, and agricultural officers stated the same. One district agricultural official, for instance, remarked that:

‘*‘even though we are informed about mining issues when we go for district assembly meetings*, *our department is not officially involved and plays no role in any of the stages regarding small-scale mining*, *be it land acquisition*, *the mining itself*, *or the land reclamation stage*, *and it’s a big worry…”*.

The current mining law (Act 703) requires that at the critical stage of site plan evaluation, MC officials should check whether the proposed mining site overlaps with an existing concession. Existing land-uses such as food and cash crops, irrigation, and farm infrastructure are not considered in licensing processes as officials rely solely on geological and concession maps to approve site plans. Despite the importance of smallholder agriculture and the many people it sustains [62, 63] the sector is not considered in the granting of ASGM mining licences. In FGDs, farmers expressed their frustration about this. A 55-year-old male cocoa farmer in Ekorso, for example, said:

*‘‘which government would go about nursing cocoa seedlings*, *farmers would go and plant and nurture them and when they grow to produce cocoa fruits*, *then some miner will show up and say the same government has given him permission to clear those cocoa trees and mine there*? *How does that help us*, *did you go* [improve] *or come* [declined]? *As a result*, *farmers are always suffering/crying within*.*”*

**Fig 6:**
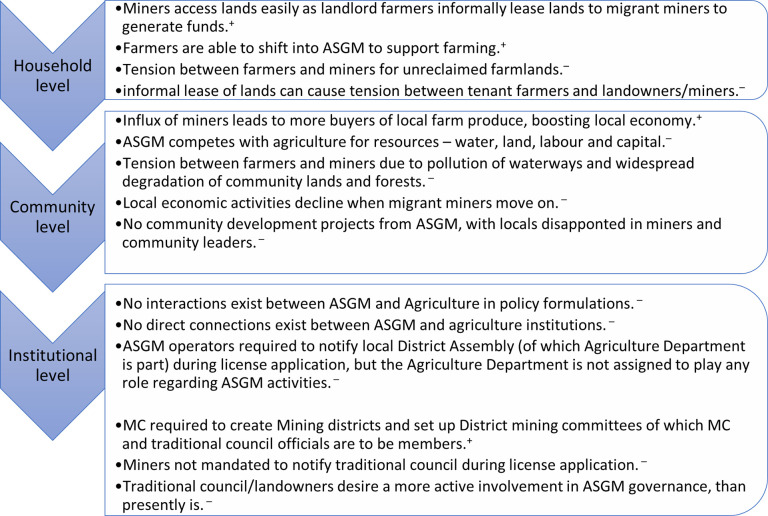
ASGM-Agriculture interactions across multiple levels. Legend: + Positive interaction;–Negative interaction. Source: Authors’ construct.

Our interviews with traditional leaders in the communities further revealed that there were no formal linkages between traditional institutions and state institutions (e.g., the District assembly and MC) regarding ASGM. Traditional institutions are not actively engaged in the governance and management of ASGM activities. Interviewees stated that they were only occasionally invited by the District Assembly to take part in an ASGM related forum, often following community level agitation. Presently, Ghana’s Act 703 (section 92) only requires that one representative of the district level traditional authority is appointed to a six-member Small-scale Mining Committee in a mining district. This committee must assist the MC District Office to monitor, promote and develop mining operations. However, these committees operate on terms and conditions determined by the Minister responsible for mining. This regulation does not meet the expectation of local traditional authorities and customary landowners, who want to be more actively engaged in ASGM governance and management (Fig 6).

The next section identifies and analyses the drivers behind the observed changes and ASGM’s interactions with farming.

### 3.2. The drivers behind interaction between ASGM, agriculture and capital

This section presents findings and discussions concerning drivers that influence the interactions between ASGM and agriculture. It touches on financial challenges of farmers, the nature of existing customary land tenure systems and informal land trading, ineffective community leadership, the mindset of miners and gold traders towards their operations, and ineffective supervision by state institutions.

#### 3.2.1. Financial challenges and hardships faced by farmers

Farmers revealed in FGDs and interviews that they faced financial hardships and lacked adequate subsidies and incentives, while simultaneously facing climate change, poor fruiting/productivity of crops due to pests and diseases, unavailable or limited markets for farm produce, and unpredictable income due to changing input prices. A district agricultural extension officer said:

‘‘*farmlands in the district are fertile*, *but farmers face multiple challenges*. *This year* [2021], *the usual rains we experience between May-July did not occur*, *cocoa plants could not fruit properly*, *and it is the end of the year now*, *and farmers report of low cocoa beans harvest due to poor fruiting*. *We think this is linked to climate change*.*”*

FGDs and interviews revealed that insufficient income from agriculture and few alternative livelihood options drove farmers to lease their land to miners, with miners promising to reclaim the lands after mining. However, most miners did not reclaim sites and farmers did not take any form of insurance against lack of reclamation. A 70-year-old male farmer from Akwabuoso explained in the FGD:

*‘*‘*Every farmer needs money*. *Maybe you can only produce 5 bags of cocoa in a year*, *yet the miner comes and offers millions for your land*, *you will collect it*! *The miner will promise you to properly reclaim the land*, *so you can have it back*. *It is because of poverty that is why we give our lands away to the miners*. *If I have money and lands*, *why will I give my land out*. *So*, *in a way due to our vulnerabilities that’s why they* [*miners*] *too had the opportunity to manipulate us in the manner they did*.*”*

Cocoa bean prices are fixed by the government, (GHS 660/US$ 88 per 64 kg bag at the time of data collection). Yet farm input prices (agrochemicals and fertilizers) and farm labour costs are unregulated and keep increasing. The government offers free annual cocoa farm spraying but farmers consider this inadequate and not all farmers benefitted from the opportunity. As such, farmers spend more money (often borrowed with high interest due to absence of any state-led credit scheme) on buying farm inputs. Nyame and Blocher (2010) [38] similarly found in Western Ghana that because of the difficulties linked to farming, landowners easily leased lands to miners because they perceived ASGM ‘‘…as an attractive and economically beneficial promise of income” (p.51).

#### 3.2.2. Customary land tenure systems and informal land markets

Customary land tenure arrangements are predominantly held over farmland in the Atiwa West area (Table 2). Interviews with the MC, Environmental Protection Agency (EPA), District Assembly, and the Lands Commission revealed that officials often blame traditional authorities and landowners for allowing ASGM and land-use conversions. A MC official at Koforidua said: ‘‘*In our view*, *chiefs and landowners are the main people responsible for the rise in ASGM*. *Outsiders cannot enter their lands and mine without their consent*.” However, FGD findings indicate that licensed miners usually approach village chiefs and landowners after licenses have been procured from the head office in Accra. They negotiate, seek permission from local chiefs and make payments before mining begins, whilst they pay compensation to farmers/local landowners for crops.

Farmers/landowners stated that concessions are granted without their consent and involvement, so miners suddenly enter their lands holding papers stating that the government granted them concessions, which causes tensions. A 60-year-old male farmer in Ekorso stated:

‘‘*When miners with concessions come*, *they first go to the palace to seek their consent and make payments*. *So*, *you will be there*, *and they will come with their machinery into your land and present some documentation as their concession from the government* [MC] *in Accra to mine*. *When you oppose*, *they don’t listen*, *your protests go nowhere*. *So*, *either you give it and get some compensation*, *or they will say*, *take them to court*.*”*

Nyame and Blocher (2010) similarly observed in Western Ghana, that landowners ‘‘…voiced strong displeasure at the legal mode of acquisition of mining rights especially relating to access to traditional lands” (p.51).

These findings show deviation from the law regarding the acquisition of an ASGM license. To acquire an ASGM licence, a prospective miner is first required to submit a site plan to the district MC for approval after identifying an area of interest (which ideally should happen with consent of the rightful landowner). Officials check this and if the proposed site does not overlap with an existing concession (ASGM or large-scale), they forward the application to Accra. In Accra, a cadastral search is undertaken and the site plan is referenced against master geologic and concession maps. If the site is free or not under a pending application and is within a designated area (i.e. not within forest reserves or buffer zones of waterbodies) MC officials allow the applicant to proceed with the remaining steps to secure a license [33, 64, 65].

Finally, licenced small-scale miners must follow compensation regulations [66]. Using baseline values including land improvements and crop ages, compensation should be calculated by ‘‘compensation committees” to provide farmers fair prices. However, interviews and FGDs revealed that formal practices tend to be replaced by informal negotiations that do not always ensure fair compensation and transparency, particularly for women and tenant farmers. Other studies have found similar outcomes [38, 63]. These frustrations also drive local landowners to exercise control over and benefit directly from the minerals in their land, despite laws on mineral rights, making it easy for illegal miners to strike direct lease deals.

While land-grabbing literature focusses on theft and dispossession [67], our findings show that complex informal land markets facilitate miners’ mineral access, reflecting dynamic relationships and practices. Hausermann et al. (2018) [63] also found this dimension of land-grabbing of artisanal miners in Western Ghana, while Van Bockstael, 2019 [68] has observed the same for Cote Ivoire. Local land-users, particularly men, are not just exploited in this system but they also actively influence land-grabbing outcomes, firmly stating their price. Mining cannot begin until landowners are satisfied with compensation. These complex land markets and negotiations result in social differentiation within communities and affect food insecurity and malnutrition [69–71] Considering the shift in blame between state officials and customary landowners, consistent and early stakeholder engagement between state officials and chiefs and residents/landowners is critical in addressing the ASGM challenges linked to current land acquisition and mining approaches.

Land-grabbing linked to informal ASGM, alongside issues of unsustainable farming practices, climate change impacts, endemic conflicts, and lack of rural employment prospects outside agriculture, particularly for the youth, also contribute to urbanisation in West Africa, affecting the region’s urban centres [72]. A well–governed ASGM sector that complements farming and allows the sustainable co-existence of both sectors would improve job opportunities in rural areas and reduce rural-to-urban migration. Critical to this is identifying productive ore deposits and demarcating ASGM zones separate from farmlands at district level. Effective ASGM zone demarcation requires the government to engage effectively with customary landowners, communities, and artisanal miners to address their varying concerns, including those bordering on mineral rights. At present, landowners’ inclination to exercise mineral rights under customary norms is driving increased illegal ASGM activity but also undermining attempts by government to formalize the ASGM sector.

#### 3.2.3. Community leadership issues

Many community leaders in new frontier areas currently lack the experience to manage the influx of miners into their spaces. Questionnaire surveys, FGDs and interviews revealed that community members were not pleased with the leadership of their chiefs and their associates. They expected them to negotiate with miners to support community developments e.g., provision of boreholes, and to restrict ASGM operations that degrade farmlands and waterbodies. Field surveys showed no developmental projects were carried out with mining funds received by traditional authorities. However, elsewhere in Ghana, Hausermann et al. (2018) found instances where mining funds were used by community leaders to build classrooms and boreholes. Informal land dealing between miners and landowners/community leaders may be convenient due to its quick and non-bureaucratic tendencies but does not guarantee direct community development.

Leaders also need to work with community members to address ASGM related challenges. Questionnaire surveys revealed that residents desired responsible ASGM practices that could coexist successfully with agriculture (Table 8). In communities where the chiefs cooperate well with their residents, they can successfully resist illegal ASGM, as reported in Asunafo–a neighbouring village to Ekorso, where *galamsey* activities do not thrive, but regulated community small-scale mining is permitted [73].

**Table 8:** Preferred changes towards ASGM activities, based on questionnaires.

Options	Percentage, n = 357
No change/do nothing	0.8
ASGM stopped outright	10.6
Progressively replaced with alternative livelihoods	5.0
ASGM kept but modified to co-exist with other livelihood activities	81.8
Other	1.7

#### 3.2.4. Attitudes of ASGM miners and gold traders

FGDs and interviews with farmers and miners showed the mindset of most miners was to ‘dig, strike, and move on’. Most miners were in a hurry and focussed on how much and how quickly they can find gold due to the high demand for it, and the pressing need to make returns on monies taken from investors/creditors. Mining gangs are in constant competition, showing little interest in related environmental and social issues. Instead of spending time reclaiming mined sites, they searched for and mined new deposits. A 49-year-old male miner in Pameng shared in an interview that:

‘‘*the high demand for gold means it is competitive out there*, *so it’s helpful to have the backing of a ‘big man’ who has political and financial influence to push for your interests*. *In some cases*, *they appoint agents to work with us on sites*.*”*

Miners linked the lack of reclamation to the non-enforcement of laws. Interviews suggested that because their colleagues do not reclaim sites, they too did not see the need. Consequently, there is little difference between the operation of the regulated and unregulated miners. This was emphasised by a 55-year-old male miner from Akwabuoso:

‘‘*I worked with one of the mining companies that ensured we were environmentally compliant*. *They would not pay a gang*, *if any member washed their dirt in the Birim river*, *plus they reclaimed sites*. *However*, *as more miners came*, *we saw chanfans on the river*, *others not reclaiming lands*, *but were not stopped/punished*. *So*, *our managers also joined*. *This led to the rampant use of inappropriate methods*, *with everyone essentially seeking as much gold as possible*.*”*

Interviews with miners revealed informal support systems in which some gold traders give interest free loans and mercury to artisanal miners that are in financial need and the miners later repay with gold. However, local gold traders did not apply any internationally recognised due diligence procedures, such as the Organization for Economic Co-operation and Development (OECD)’s Due Diligence Guidance. They showed little interest in whether the miners they buy from operate legally and responsibly [74]. Gold traders were preoccupied with ensuring they were not outwitted by ‘dodgy’ gold sellers (who sometimes sell gold samples embedded with stone/other metals). Some traders demanded the mercury-gold amalgam to be heated in their presence before making payments.

#### 3.2.5. Ineffective monitoring and support from relevant institutions

Weak enforcement of laws and regulations drive degradation. Farmers in FGDs complained of insufficient assistance from state agencies such as the MC, EPA, District Assembly, and the police, in dealing with miners that have degraded lands (also see Table 9). Farmers argued that miners often paid their way out of trouble. Because miners have more money than the farmers, and sometimes political backing, it was difficult to challenge them or engage in legal disputes. A 47-year-old male cocoa farmer in Ekorso stated:

*‘‘We see government personnel mandated to supervise the miners come around with police and do their rounds but*, *in the end*, *the miners will continue to work in bad ways*, *with some not having the correct paperwork*. *If you report your case to them*, *you get no good treatment*, *and it gets nowhere*.”

This highlights the increasing power imbalance between the more powerful ASGM entrepreneurs (with more money) and the relatively powerless smallholder farmers.

**Table 9:** Residents’ perceived levels of organisational support, based on questionnaire survey. Units are percentages.

	Farmer association	Local mining association	Community social support association	Local traditional council	Local district assembly/other government agencies
**Not at all**	53.7	87.2	56.8	50.0	48.6
**Low**	11.6	4.3	10.3	4.9	2.7
**Medium**	28.9	7.0	23.2	7.1	7.0
**High**	5.3	1.1	9.2	24.7	16.8
**Very high**	0.5	0.5	0.5	13.2	24.9

A 58-year-old male cocoa farmer in an FGD in Ekorso stated:

‘‘*When our farm road got damaged and our chief farmer and other elders informed the district police commander*, *he said he would come and see it*, *but nothing happened*. *With such an outcome*, *what can you do*? *Do you have the money to pursue a legal case with the miners*?*”*

Effective supervision of ASGM by state agencies is compromised by the interest and interference of politicians, as well as miners’ financial capabilities and interests to pay their way out of trouble. Farmers feel helpless and increasingly overwhelmed, which can increase confrontations if the trend continues. Lack of MC and EPA district offices in new mining areas such as our study area compound law enforcement challenges. Interviews with MC and EPA officials revealed that they do not have enough personnel and resources to adequately monitor ASGM in all locations, with MC officers adding that they were now in the process of creating a mining district in Atiwa West.

### 3.3. Policy implications of agriculture and ASGM interactions

Existing literature on ASGM-agriculture linkages has centred on exploring how the two practices interact on the ground and how farming and mining households adapt consequently. ASGM and agriculture have been described by others as complementary rural poverty-reducing activities, focusing on the individual/household level, including a fragile co-existence found in Peru [10, 30, 31]. The novelty of this study is that it has explored ASGM-agriculture interaction at multiple levels–household, community, and institutional levels–in a frontier area where ASGM is growing. These interactions are discussed in the subsections next.

#### 3.3.1. Household level

Our findings indicate that ASGM activities can be beneficial to its practitioners and can support agricultural activities at the household level but that the links between ASGM and agriculture are not entirely complementary. To foster household level complementarity, MC should link with the agricultural department and other relevant stakeholders including District Assemblies, traditional authorities, and the EPA, to deal with land degradation risks by: i) Adopting land degradation preventative approaches: educate farmers to be weary of irresponsible mining practices and insure in case of miners’ lack of interest in reclaiming lands; train miners to adopt responsible mining practices; support farmers to form or join functional farmer-based cooperatives; and train farmers/landowners on valuation and negotiation skills that enable them to demand appropriate compensation for crops and lands; ii) Tackling improperly reclaimed lands: agricultural officers should train affected farmers in land-crop suitability and guide them to plant suitable crops e.g., oil palm that can enhance stability of soils and stimulate faster recovery of soil nutrients over time, as well as ensuring farmers are provided with an overview of the longer-term risks associated with planting particular crops.

Additionally, the agricultural sector, including Ghana Cocoa Board, needs to introduce policies that protect the interests of farmers, such as an insurance scheme that insulates farmers from forceful eviction from lands by landowners wishing to lease lands to miners. Farmer associations need to be strengthened to better advocate for farmers in the face of growing ASGM activity. Government must similarly take steps to make farming, including cocoa cropping, more lucrative and attractive by: i) educating and building the capacities of smallholders to shift from unsustainable farming practises and improve their agribusiness skills; ii) providing more support to farmers through regular supply of adequate farm inputs e.g, improved seeds at affordable prices and iii) providing adequate financial, logistical, and technical support for increased profitability.

Miners in the ASGM sector struggle to obtain investment finance yet they must still front the costs for land reclamation. Regulation 23 of (L.I 1652) [75] mandates the EPA to ensure that prospective small-scale miners post reclamation bonds. Policy considerations regarding costs and financing are necessary. Illegal miners have easy access to the gold market because licensed gold traders buy from both legal and illegal miners. Regulations and policies need updating to ensure that more artisanal miners are motivated to register and operate responsibly so that they can complement rather than undermine agricultural activities. For instance, gold traders could be empowered and supported by the state to buy gold at higher prices from registered and certified responsible miners.

#### 3.3.2. Community level

The itinerant nature of miners and the irresponsible mining practices the majority adopt mean that benefits obtained from ASGM at individual or household level are undermined by aggregate long-term costs at community level linked to degradation of physical capitals and natural capitals such as farmlands, water resources and forests. Our study highlights important aspects of gold mining activities that are related to agriculture, such as land tenure conflicts, land reclamation and land grabbing. The Ghanaian government launched a national land reclamation project in 2021 [76] targeting abandoned unreclaimed lands. This needs to incorporate innovative community-based reclamation processes that are economically affordable, socially acceptable, and ecologically viable [77]. In Indonesia, community participation in land reclamation projects proved very successful [78]. Community leadership and farmers’ cooperatives need to be empowered on ways to handle the influx of ASGM operators and related social conflicts between ASGM and agriculture.

#### 3.3.3. Institutional level

Our findings again revealed a lack of institutional linkages between the mining and agricultural sectors. It shows that without intervention, ASGM and agriculture are far from complementary rural poverty-reducing activities. A key proposition of this paper is that sustainable and mutually-beneficial interactions between agriculture and ASGM activities on the ground require interactive linkages among relevant state and non-state institutions within the agricultural, mining, land and local authority sectors, functioning within an effective dynamic legal and regulatory setting to enforce responsible ASGM as well as sustainable agricultural activities.

Ghana’s mining legal framework only mandates the mineral rights holder to negotiate with and compensate the landholder before mining operations begin. These negotiations and compensations are, however, centred on an economic valuation of land use activities on the land [66]. Compensation procedures do not consider any costs linked to potential post-mining land use challenges, or potential impacts of mining pollution on health of residents. Similarly, landowners do not get to share directly in the proceeds of gold mined from their lands. The growth of informal ASGM shows that there is a disconnect between mining legislation and the realities on the ground.

Ghana’s existing ASGM formalisation regime is strictly state-centred, and customary land tenure rights are not effectively incorporated. Most small-scale miners choose to disregard state laws and deal exclusively with customary landowners to access land and mine informally. Currently there are few incentives for landowners to engage in ASGM formalization processes, causing landowners to approve miners to mine illegally. Formalization of ASGM is a global challenge, and this study prompts the urgent need for states to closely consider customary land tenure rights and interests in ASGM formalization processes.

The findings from this study, however, also point to a problem with customary laws. Many local and traditional leaders abuse their power, and this reinforces socio-economic differentiations and inequalities. Therefore, mineral-endowed nations in the Global south and particularly in SSA, need to move away from state-centric formalisation to integrating customary laws in ASGM formalisation processes. States should engage with traditional leaders and landowners to collaborate and map out a hybrid customary and statutory governance approach. It should support environmentally responsible and socio-economically viable ASGM activities that will enhance agriculture and rural development and allow landowners and local and traditional authorities to share in ASGM benefits (also see [34]).

Through effective collaboration with traditional authorities and customary landowners, governments should be committed to proactively prioritise geological assessments and block out mineral rich lands for ASGM to limit interference with agricultural productivity and other relevant land use activities such as large-scale mining. The MC and EPA should adopt strict monitoring and enforcement of laws that lead to responsible mining operations and reclamation of mined lands, ensuring reclamation works are verified and certified, taking appropriate action when expected reclamation standards are not met.

**Fig 7:**
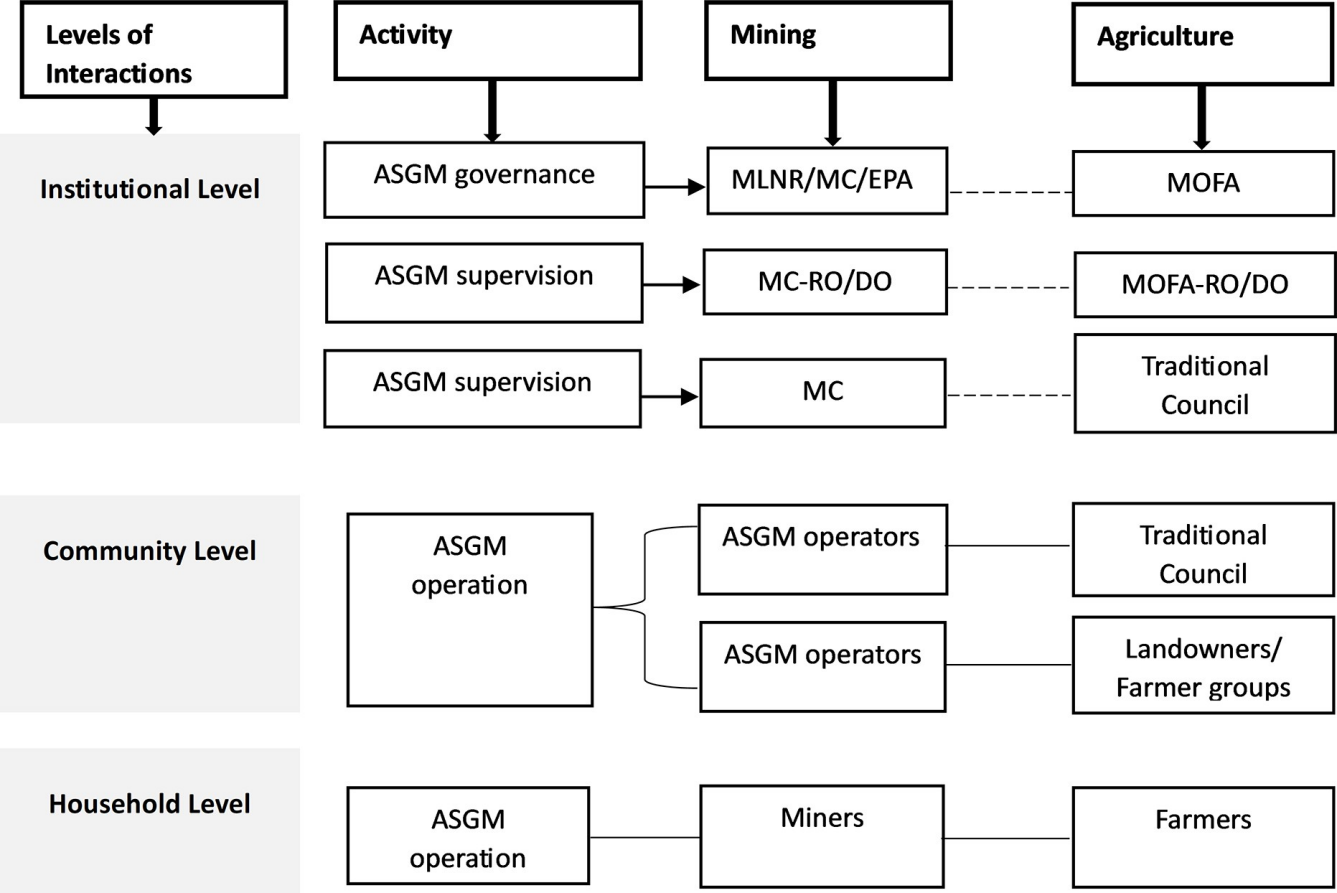
ASGM-Agriculture interactions across institutions. --------- *No direct connection between sectors*. __________ *Direct connection exists between sectors*. *MLNR – Ministry of Lands and Natural Resources; MOFA – Ministry of Food and Agriculture*. *MC – Minerals Commission; EPA—Environmental Protection Agency*. *RO – Regional office; DO – District Office*. *Source*: *Authors’ construct*.

Our findings have shown how closely the agricultural and ASGM sectors are linked in practice and have revealed some of the many factors that drive, shape, and explain their relationship. But these linkages are not reflected in policy and governance in Ghana, as is the case for some other African countries [27]. In Ghana, each sector has traditionally developed policies independent of the other and there is no formal district, regional and national level institutional coordination between the two sectors (Fig 7). ASGM, when regulated and practised sustainably, can effectively co-exist with cocoa farming and other agricultural practices. There is a pressing need for a joined-up governance framework that sees ASGM and agriculture as interconnected activities rather than as livelihood alternatives [57].

A more comprehensive rural development policy stimulating sustainable enhancement of the two livelihood options is necessary. Policies and regulations should demand effective stakeholder engagement regarding acquisition of lands and ensure responsible practices in both livelihood activities. Stakeholders should include the government agencies on mining especially the MC, the agricultural ministry, customary landowners and traditional councils, farmers and their unions/associations, miners and their associations, the local government ministry and district assemblies, government land agencies, EPA, and Water Resources Commission, amongst others.

## 4. Conclusion

This study has shown that ASGM emergence boosts local economic activities and triggers movement of labour and finance across ASGM and agriculture sectors at household level. ASGM is not replacing farming in non-traditional mining areas but adds to the existing rural livelihood mix. However, most miners are unregulated entrepreneurs and mine irresponsibly, degrading natural capital such as farmlands, waterbodies, and forests, as well as physical resources like farm roads. Miners generally do not undertake community development projects. This relationship between ASGM and agriculture is unsustainable because irresponsible itinerant ASGM is creating tensions between farmers and miners. While ASGM generates short–term income at an individual and household scale for some, benefits are outweighed by the long–term costs of ASGM at the community scale, linked to the aggregate degradation and loss of agricultural lands, soil contamination, and pollution of waterways and the atmosphere.

Low profitability in farming drives landowners to lease lands to miners and generate funds to enhance livelihoods, causes youth to move into mining full time, and leads farmers to diversify into ASGM and raise funds to support farming. Lack of law enforcement, absence of appropriate incentives and ineffective stakeholder engagement and the mindset of many miners and investors to maximize profit quickly also drive informal ASGM. Government’s inconsistent engagement with landowners and traditional leaders and inadequate compensation to landholders for mining sites, drives landholders into informal direct lease deals with miners to exercise control.

ASGM interacts with agriculture in communities but in governance the two are not considered simultaneously. Our study of ASGM in newly mined areas shows that without intervention, ASGM and agriculture are far from complementary rural poverty-reducing activities. There is an urgent need for more joined–up governance both across the sectors and between formal and informal (traditional) institutions.

Future studies could assess human capital impacts from the mining, drawing on expertise from the health sciences. Food quality tests of farm produce from reclaimed lands could be carried out in future to test if they are appropriate for human consumption. Additionally, policy alignment between mining and agriculture could be assessed.

## Supporting information

S1 Appendix Appendix A—Descriptive statistics output from questionnaire surveys. (DOCX)

S1 File Household questionnaire. (DOCX)

S2 File Focus group discussion template. (DOCX)

S3 File Oral history template. (DOCX)

S4 File Environmental inventory & transect walk checklist. (DOCX)

S5 File Sources of public datasets used. (DOCX)
