# Supplementary material for: Joined-up governance for more complementary interactions between expanding artisanal small-scale gold mining and agriculture: Insights from Ghana
Source: PLoS One. 2024 Apr 4;19(4):e0298392. doi: 10.1371/journal.pone.0298392 (PMC10994392; doi:10.1371/journal.pone.0298392)
Supplement: S1 Appendix — (DOCX) [file pone.0298392.s001.docx]

**Appendix A**

**Descriptive statistics output (frequency distribution and mean) from questionnaire survey data**

**Agriculture:**

1. **Percentage of farmers into cocoa farming**

| Total number of farmers | Number of Cocoa farmers | Percent of cocoa farmers (174/219*100) |
| --- | --- | --- |
| 219 | 174 | 80% |

1. **How long have you been engaged in farming?**

| Category | Frequency | Percent | Valid Percent | Cumulative Percent |
| --- | --- | --- | --- | --- |
| <2 years | 2 | 0.6 | 0.9 | 0.9 |
| 2-5 years | 14 | 3.9 | 6.4 | 7.3 |
| 6-10 years | 24 | 6.7 | 11.0 | 18.3 |
| 11-20 years | 53 | 14.7 | 24.2 | 42.5 |
| >20 years | 126 | 35.0 | 57.5 | 100.0 |
| Total | 219 | 60.8 | 100.0 |  |

1. **What is the size of the farmland your household regularly cultivate (average cultivated land for the past 3 years in acres)? - Both (cash and food)**

| Crop | N | Minimum | Maximum | Mean (acres) | Std. Deviation |
| --- | --- | --- | --- | --- | --- |
| For both cash and food crops | 139 | 1 | 40 | 7.07 | 6.132 |
| Cocoa | 174 | 0 | 49 | 5.72 | 5.250 |

1. **Mean size of total farmland held by each household:**

Case Processing Summary

|  | Valid | | Cases Missing | | Total | |
| --- | --- | --- | --- | --- | --- | --- |
|  | N | Percent | N | Percent | N | Percent |
| Total farmland size per each farmer | 219 | 60.8% | 141 | 39.2% | 360 | 100.0% |

Descriptives

|  |  |  | Statistic | Std. Error |
| --- | --- | --- | --- | --- |
| Total farmland size per each farmer | Mean |  | 6.76 | 0.437 |
|  | 95% Confidence Interval for Mean | Lower Bound | 5.90 |  |
|  |  | Upper Bound | 7.62 |  |
|  | 5% Trimmed Mean |  | 5.99 |  |
|  | Median |  | 5.00 |  |
|  | Variance |  | 41.770 |  |
|  | Std. Deviation |  | 6.463 |  |
|  | Minimum |  | 0 |  |
|  | Maximum |  | 49 |  |
|  | Range |  | 49 |  |
|  | Interquartile Range |  | 6 |  |
|  | Skewness |  | 2.762 | 0.164 |
|  | Kurtosis |  | 11.709 | 0.327 |

1. **How many of the following items did your household own or harvested in the previous agricultural season?**

**Descriptive Statistics**

| Crop (bag size is 100kg, except cocoa = 64kg) | N | Minimum number of bags | Maximum number of bags | Mean number of bags | Std. Deviation |
| --- | --- | --- | --- | --- | --- |
| Cocoa | 172 | 0 | 70 | 9.41 | 9.283 |
| Oil palm | 12 | 1 | 120 | 31.25 | 40.118 |
| Maize | 128 | 0 | 30 | 5.21 | 4.494 |
| Plantain | 127 | 1 | 200 | 14.24 | 21.790 |
| Cassava | 144 | 0 | 54 | 8.56 | 9.728 |
| Yam | 6 | 2 | 15 | 5.67 | 4.803 |
| Cocoyam | 29 | 1 | 40 | 4.97 | 7.528 |

1. **Do you have access to credit or loans for your agricultural activities?**

|  |  | Frequency | Percent | Valid Percent | Cumulative Percent |
| --- | --- | --- | --- | --- | --- |
| Valid | No | 191 | 53.1 | 87.2 | 87.2 |
|  | Yes | 28 | 7.8 | 12.8 | 100.0 |
|  | Total | 219 | 60.8 | 100.0 |  |
| Missing | System | 141 | 39.2 |  |  |
| Total | 360 | 100.0 |  |  |  |

**ASGM:**

1. **Approaches to ASGM operations**

| Operational type | Frequency | Percent frequency |
| --- | --- | --- |
| ASGM only on permanent basis | 37 | 31.36 |
| ASGM mainly with some farming elsewhere | 12 | 10.17 |
| Shifts between farming and ASGM seasonally in the community | 43 | 36.44 |
| Just as investor (do not physically take part) | 2 | 1.69 |
| Other (does non-farm job with mining as a support) | 24 | 20.34 |
| Total | 118 | 100 |

From table above, number of farmer-miners = 12+43 = 55

Proportion of farmers combining farming and mining = 55/219 * 100 = 30.59 = 30.6%.

1. **If applicable, how many members of your household undertake ASGM activities, what are their ages?**

| Age group (yrs) | Frequency |
| --- | --- |
| 18 | 8 |
| >18-30 | 53 |
| 31-40 | 41 |
| 41-50 | 11 |
| >51 | 5 |

1. **How miners acquired farmland**

| Ways miners acquire farmland | frequency | Percentage frequency (%) |
| --- | --- | --- |
| Purchase from farmer | 21 | 23.59 |
| Purchase from landowner | 44 | 49.44 |
| Resource exchange/barter trade | 0 | 0.0 |
| Invasion | 24 | 26.97 |
| Total | 89 | 100.00 |

1. **If applicable, which ways do you get access to credit/loans for your mining activities?**

| Means of access | Frequency |
| --- | --- |
| Family/friends | 21 |
| Bank/other financial institutions | 1 |
| private persons in ASGM/’middlemen’ | 4 |
| private business | 0 |
| NGOs | 0 |
| Community based organization | 0 |
| Other | 2 |

1. **Has your farmland ever been degraded or lost through ASGM activity?**

|  |  | Frequency | Percent | Valid Percent | Cumulative Percent |
| --- | --- | --- | --- | --- | --- |
| Valid | No | 137 | 38.1 | 62.3 | 62.3 |
|  | Yes | 83 | 23.1 | 37.7 | 100.0 |
|  | Total | 220 | 61.1 | 100.0 |  |
| Missing | System | 140 | 38.9 |  |  |
| Total |  | 360 | 100.0 |  |  |

1. **Size of degraded farmland per household**

Case Processing Summary

|  | Valid | | Cases Missing | | Total | |
| --- | --- | --- | --- | --- | --- | --- |
|  | N | Percent | N | Percent | N | Percent |
| Total size of farmland degraded | 80 | 22.2% | 280 | 77.8% | 360 | 100.0% |

**Descriptives**

|  |  |  | **Statistic** | **Std. Error** |
| --- | --- | --- | --- | --- |
| Total size of farmland degraded | Mean |  | 2.17 | 0.222 |
|  | 95% Confidence Interval for Mean | Lower Bound | 1.72 |  |
|  |  | Upper Bound | 2.61 |  |
|  | 5% Trimmed Mean |  | 1.94 |  |
|  | Median |  | 1.75 |  |
|  | Variance |  | 3.929 |  |
|  | Std. Deviation |  | 1.982 |  |
|  | Minimum |  | 0 |  |
|  | Maximum |  | 9 |  |
|  | Range |  | 9 |  |
|  | Interquartile Range |  | 1 |  |
|  | Skewness |  | 1.886 | 0.269 |
|  | Kurtosis |  | 3.069 | 0.532 |

1. **Percentage of farmland degraded by ASGM per household**

| Total farmland size per household – mean (std dev) | Total farmland size degraded by ASGM per household – mean (std dev) | Percent size of farmland degraded. |
| --- | --- | --- |
| 6.76 (6.463) | 2.17 (1.982) | 32.1% |

1. **In your estimation, to what extent has ASGM impacted your access to farm labour?**

|  |  | Frequency | Percent | Valid Percent | Cumulative Percent |
| --- | --- | --- | --- | --- | --- |
| Valid | Increased by | 18 | 5.0 | 8.4 | 8.4 |
|  | decreased by | 151 | 41.9 | 70.2 | 78.6 |
|  | No change | 27 | 7.5 | 12.6 | 91.2 |
|  | Not sure | 19 | 5.3 | 8.8 | 100.0 |
|  | Total | 215 | 59.7 | 100.0 |  |
| Missing | system | 145 | 40.3 |  |  |
| Total |  | 360 | 100.0 |  |  |

1. **To what extent has the cost of hiring farm labourers changed due to the rise of ASGM?**

|  |  | Frequency | Percent | Valid Percent | Cumulative Percent |
| --- | --- | --- | --- | --- | --- |
| Valid | Increased by | 170 | 47.2 | 78.7 | 78.7 |
|  | decreased by | 5 | 1.4 | 2.3 | 81.0 |
|  | No change | 20 | 5.6 | 9.3 | 90.3 |
|  | Not sure | 21 | 5.8 | 9.7 | 100.0 |
|  | Total | 216 | 60.0 | 100.0 |  |
| Missing | System | 144 | 40.0 |  |  |
| Total |  | 360 | 100.0 |  |  |

1. **How has the price of food been impacted since the rise of ASGM began?**

|  |  | Frequency | Percent | Valid Percent | Cumulative Percent |
| --- | --- | --- | --- | --- | --- |
| Valid | Increased by | 284 | 78.9 | 80.5 | 80.5 |
|  | decreased by | 9 | 2.5 | 2.5 | 83.0 |
|  | No change | 47 | 13.1 | 13.3 | 96.3 |
|  | Not sure | 13 | 3.6 | 3.7 | 100.0 |
|  | Total | 353 | 98.1 | 100.0 |  |
| Missing | System | 7 | 1.9 |  |  |
| Total |  | 360 | 100.0 |  |  |

1. **What changes will you like to see regarding the ASGM activities in your community?**

|  |  | Frequency | Percennt | Valid Percent | Cumulative Percent |
| --- | --- | --- | --- | --- | --- |
| Valid | No change/do nothing | 3 | 0.8 | 0.8 | 0.8 |
|  | Stopped outright | 38 | 10.6 | 10.6 | 11.5 |
|  | progressively replaced with alternative livelihoods | 18 | 5.0 | 5.0 | 16.5 |
|  | Kept but modified to co-exist with other livelihood options | 292 | 81.1 | 81.8 | 98.3 |
|  | other | 6 | 1.7 | 1.7 | 100.0 |
|  | Total | 357 | 99.2 | 100.0 |  |
| Missing | System | 3 | 0.8 |  |  |
| Total |  | 360 | 100.0 |  |  |
