# Supplementary material for: Joined-up governance for more complementary interactions between expanding artisanal small-scale gold mining and agriculture: Insights from Ghana
Source: PLoS One. 2024 Apr 4;19(4):e0298392. doi: 10.1371/journal.pone.0298392 (PMC10994392; doi:10.1371/journal.pone.0298392)
Supplement: S1 File — (DOCX) [file pone.0298392.s002.docx]

**HOUSEHOLD QUESTIONNAIRE**

Enumerator’s initials __________ Name of village____________ Interview date___________

Select where applicable and please provide answers to open ended questions.

**About you and your household**

1. What is your gender?

1. Male 2. Female 3. Prefer not to say

2. Please indicate your age category from the options below.

All interviewees must be 18 years or above

1. 18 to 24

2. 25 to 34

3. 35 to 44

4. 45 to 54

5. 55 to 64

6. 65 to 74

7. 75 or older

3. How long have you lived in this community?

_____years

4. a) Are you local or do you come from somewhere else?

1. Indigene 2. Migrant 3. Other

b) If you are a migrant, what is your home region/country __________

5. How would you describe the status of your home?

1. Own

2. Renting - Private tenant

3. Family home

4. Other

5. Prefer not to say

6. Which type of house do you live in?

1. house roofed with zinc/aluminium sheets or slate

2. house roofed with thatch or related material.

7. How would you describe your marital status:

1. Single

2. Married

3. Divorced

4. Separated

5. Co-habiting/Partner

6. Widowed

8. Are you the head of the household (household is defined by those who eat from the same pot)?

1. Yes 2. No

9. If no, who is the head of the household?

1. Husband/male partner

2. Wife/female partner

3. Other ______

4. not applicable

10. Where is the head of the household permanently residing (> 50% of the time)?

1. In this village/With me

2. Another village

3. Another town/city

4. Another country

11. What is the highest level of education completed by the household head?

1. None

2. Primary school

3. Junior high school (middle school)

4. Senior High school/A-level

5. Other vocational/technical training college

6. Bachelor’s degree or equivalent

7. Higher degree or equivalent

8. Other (e.g., non-formal education)

12. How many people currently live in your household?

| 1.Male adults (>18years) |  |
| --- | --- |
| 2.Female adults (>18 years) |  |
| 3.Male children (< 18 years) |  |
| 4.Female children (<18years) |  |
| 5.Total |  |

**Wealth ranking (household vulnerability assessment)**

13. Which of the following sources do your household engaged in as means of making a living? Please assign percentage income to each of the activities you undertake.

| Source of livelihood | Percentage income contribution (%) |
| --- | --- |
| 1. Cash crop cultivation (e.g., cocoa, oil palm, citrus) |  |
| 2. Arable crop cultivation (e.g., maize, plantain, cassava, vegetable, etc) |  |
| 3. Livestock |  |
| 4. Forest resource exploitation *(non-timber - e.g., hunting, harvesting of leaves, climbers & others)* |  |
| 5. Gold Mining (small-scale) |  |
| 6. Other small-scale mining (diamond, kaolin, sand, aggregates, etc) |  |
| 7. Professional (*large-scale mining employee, clerical officer, teacher/gov’t worker, religious worker, etc*) Specify _____ |  |
| 8. Waged labour  *(e.g., farm employee, aquaculture employee, construction labourer, other)* |  |
| 9. Industry  *(e.g manufacturing /processing, e.g., saw milling, basket weaving, wood carving, corn milling, palm oil produce, etc)* |  |
| 10. Trading in agricultural produce (cocoa buyer, other farm produce, fish etc) |  |
| 11. Service  *(e.g. shop, taxi/driver, food vending/ restaurant, rental, artisanship etc.)* |  |
| 12. Fishing (from natural waters*)* |  |
| 13. Aquaculture (pond owner) |  |
| 14. Retiree on pension |  |
| 15. Migration/remittances |  |
| 16. Other__________________ |  |
| Total | 100% |

14. How many of the following items did your household own or harvested in the previous agricultural season?

| Item | numbers harvested/possessed |
| --- | --- |
| 1.bag of cocoa |  |
| 2.bag of oil palm |  |
| 3.bag of citrus/orange |  |
| 4.bag of cashew |  |
| 5.bag of maize |  |
| 6.bag of plantain |  |
| 7.bag of cassava |  |
| 8.bag of yam |  |
| 9.bag of rice |  |
| 10.bag of cocoyam |  |
| 11.bag of beans |  |
| 12.Pineapple |  |
| 13.bag of vegetable (specify______) |  |
| 14. bag of fruit (specify______) |  |
| 15.Cattle |  |
| 16.Sheep and goat |  |
| 17.poultry |  |
| 18.other_____________ |  |

15 Could you please answer yes or no to whether you own any of the following items?

| Item | Yes | No |
| --- | --- | --- |
| TV |  |  |
| Radio |  |  |
| Mobile phone |  |  |
| Internet in the home |  |  |
| Car/truck |  |  |
| Motorbike/motor tricycle |  |  |
| Bicycle |  |  |
| Motor boat |  |  |
| Manual boat |  |  |
| Crop sprayer |  |  |
| Other |  |  |

16 Which of the items do you need in order to pursue your activity that generates the most income?

| Item | Needed |
| --- | --- |
| TV |  |
| Radio |  |
| Mobile phone |  |
| Internet in the home |  |
| Car/truck |  |
| Motorbike/motor tricycle |  |
| Bicycle |  |
| Motor boat |  |
| Manual boat |  |
| Crop sprayer |  |
| Other |  |

17. How easy it is to feed your household and provide educational support?

Very easy =1, to very difficult = 5

Very easy easy moderate difficult very difficult

18. In the event where a member of your household requires hospital treatment how easily could you arrange transport and other resources needed to care for the member? Very easy =1 to very difficult = 5

Very easy easy moderate difficult very difficult

**Characteristics of farming**

19. if you are a farmer, which type of farming do you mainly practice? If not move to 33.

1. arable cropping

2. cash cropping

3. both (arable & cash)

4. livestock

5. mixed (livestock & crop)

6. Other

7. Not applicable

20. How long have you been engaged in farming?

1. <2 years 2. 2-5 years 3. 6-10 years 4. 11-20 years 5. >20 years

21. What kind of tenure arrangement do you hold over the farmland? Select all that apply.

1. owner (inherited)

2. owner (private purchase)

3. owner (gifted)

4. rented

5. share cropping

6. caretaker

7. other

22. What is the size of the farmland your household regularly cultivate (average cultivated land for the past 3 years in acres)? (smallholder <3ha; large holder 3ha & above). Please key actual figure and select correct range too.

| Farming type | Size (acres) | 1. < 5 acres | 2. 5 - 10 acres | 3. 11 - 15 acres | 4. 16–20 acres | 5. >20 acres |
| --- | --- | --- | --- | --- | --- | --- |
| Arable crop |  |  |  |  |  |  |
| Cash crop |  |  |  |  |  |  |
| Both (cash and food) |  |  |  |  |  |  |
| Livestock |  |  |  |  |  |  |
| total |  |  |  |  |  |  |

23. If you are into cash crop farming, how many acres of land do your household cultivate for any of the following crops? Please key actual figure and select correct range too.

|  | Cocoa | oil palm | citrus | cashew | other (specify) |
| --- | --- | --- | --- | --- | --- |
| Size in acres |  |  |  |  |  |
| 1. < 2 acres (0.7 ha) |  |  |  |  |  |
| 2. 2-5 acres (0.8-2 ha) |  |  |  |  |  |
| 3. 5-8 acres (2.1-3.2 ha) |  |  |  |  |  |
| 4. 8–10 acres (3 – 4 ha) |  |  |  |  |  |
| 5. 10-13 acres (4.1-5.2 ha) |  |  |  |  |  |
| 6. > 13 acres (>5.2 ha) |  |  |  |  |  |

24. Do you engage the services of paid labourers on your farm(s)?

1. Yes 2. No

25. Do you have access to credit for your agricultural activities?

1. Yes 2. No

25 (b) If yes, from which source(s)?

1. Private enterprise
2. Bank
3. Government
4. Non-governmental organisation
5. Community-based organisation
6. Family/friend
7. other

**Conflicting relationships between ASGM and agriculture**

26. Has your farmland ever been degraded or lost through ASGM activity?

1. Yes 2. No

27. If yes, what is the approximate size of farmland that was degraded through ASGM?

If no, please go to question 34.

| Farm type | Size (acres) |
| --- | --- |
| 1. arable crop |  |
| 2. cash crop |  |
| 3. both (cash & arable |  |
| 4. fallow field |  |
| 5. livestock |  |
| 6. other |  |
| 7. total |  |

28. If you are into cash crop farming, how many acres of land was approximately degraded through ASGM activities? Please select as many as applicable

1. Cocoa _______2. oil palm ________3. citrus _______ 4. cashew_______ 5. other_____

29. How did the miners acquire your farmland?

1. purchase from farmer

2. purchase from landowner

3. resource exchange/barter trade

4. forceful access/invasion

5. other

30. How has your farmland been affected? (Select as many as applicable)

1. Loss of farmland resulting from actual mining

2. Degraded portions due to informal routes created by miners

3. Degraded portions due to temporal accommodation pitched by miners

4. Overflow of polluted water into farmland

5. Destruction of crops

6. forceful harvesting of crops for use by miners

7. Other __________

31. In your estimation, to what extent has ASGM impacted your access to farm labour?

Please state in percentage.

1. increased by ______% 2. decreased by _______% 3. no change 4. not sure

32. To what extent is the cost of hiring farm labourers changed due to the rise of ASGM?

Please state in percentage.

1. increased by _____% 2. decreased by _____% 3. no change 4. not sure

33. How has the price of food been impacted since the rise of ASGM began?

Please state in percentage.

1. increased by _____% 2. decreased by _____% 3. no change 4. not sure

34. Do you encounter any problem in terms of the following due to ASGM activities and at what level of impact? 1 = very low impact, 5 = very high. *select all that is applicable.* (Please select one option per line).

|  | Very low | Low | Medium | High | Very high |
| --- | --- | --- | --- | --- | --- |
| Conflicts with landowners |  |  |  |  |  |
| High cost of input equipment/chemicals/Seeds |  |  |  |  |  |
| Poor accessibility of roads to farm |  |  |  |  |  |
| Conflicts with ASGM miners? |  |  |  |  |  |

35. How would you score the importance of each of these factors in terms of their influence on conflicts between agriculture and ASGM? 1 = very low influence, 5 = very high.

(Please select one option per line).

|  | Very low | Low | Medium | High | Very high |
| --- | --- | --- | --- | --- | --- |
| Leadership issues in the community |  |  |  |  |  |
| Land tenure arrangements |  |  |  |  |  |
| Behaviour of landowners |  |  |  |  |  |
| Attitudes of the small-scale miners |  |  |  |  |  |
| Behaviour and influence of politicians |  |  |  |  |  |
| Behaviour of gold traders/ ‘middlemen’ |  |  |  |  |  |
| Influence from local assembly |  |  |  |  |  |
| Lack of support from government agencies |  |  |  |  |  |
| nature of existing laws |  |  |  |  |  |
| Ways mining policies are being implemented |  |  |  |  |  |

**Characteristics of ASGM**

36. if applicable, how many members of your household undertake ASGM activities, what are their ages and how long have they been involved? If not applicable, please move to 45.

| age | Male | female | Years involved |
| --- | --- | --- | --- |
| <18 |  |  |  |
| 18-30 |  |  |  |
| 31-40 |  |  |  |
| 41-50 |  |  |  |
| >51 |  |  |  |

37. In which manner is the ASGM operation carried out?

1. ASGM only on permanent basis

2. ASGM mainly with some farming elsewhere

3. Shifts between farming and ASGM seasonally in the community

4. Just as investor (do not physically take part)

5. other

38. If you are into mining on permanent basis, what was your previous job?

1. farming related activities 2. non-farm activities 3. Not applicable

39. If applicable, which ways do you get access to credit/loans for your mining activities?

1. family/friends

2. bank/other financial institutions

3. private persons in ASGM/’middlemen’

4. private business

5. NGOs

6. community-based organisations

7. other

8. not applicable

40. How easy is it for miners to acquire requisite lands for ASGM activities in your community?

1 = very easy, 5 = very difficult.

1. very easy 2. easy 3. somewhat difficult 4. difficult 5. very difficult

41. Approximately how much revenue (gross income) does your household generate from ASGM each year?

______________

42. What factors influence miners not to reclaim mine sites or be environmentally compliant? (not at all = 1 to very high = 5) (Please select more than one if applicable).

|  | Not at all | Low | Moderate | High | Very high |
| --- | --- | --- | --- | --- | --- |
| Money (cost) |  |  |  |  |  |
| Time and effort |  |  |  |  |  |
| Lack of abilities/skills |  |  |  |  |  |
| Lack of knowledge/awareness |  |  |  |  |  |
| Attitudinal issues (bad mindset) |  |  |  |  |  |
| Other (specify) _____ |  |  |  |  |  |

**Complementary relationships between ASGM and agriculture**

43. About what percentage of your household ASGM earnings is used to support farming each year?

________%

44. How much of your household farming revenue, if any, is used to boost ASGM activities in each year?

________%

45. How willing are you to hire or sell your farmland to ASGM entrepreneurs, because of the monetary proceeds?

1.Not at all 2. Uncertain 3. Somewhat willing 4. Willing 5. Very willing

46. Have you ever had your land reclaimed properly after ASGM activity?

1. Yes 2. No 3. Not applicable

47. When mine sites are properly reclaimed, what effects do they have on agriculture production afterwards.

1. enhance productivity

2. decrease productivity

3. no change

4. not sure

5. not applicable

48. How would you score the importance of each of these factors in terms of their influence on co-existence between agriculture and ASGM? 1 = very low, 5 = very high.

(Please select one option per line).

|  | Very low | Low | Medium | High | Very high |
| --- | --- | --- | --- | --- | --- |
| Leadership in the community |  |  |  |  |  |
| Land tenure arrangements |  |  |  |  |  |
| Positive attitude of landowners |  |  |  |  |  |
| Positive attitudes of the miners |  |  |  |  |  |
| Behaviour and influence of politicians |  |  |  |  |  |
| Influence from local assembly |  |  |  |  |  |
| Support from government agencies |  |  |  |  |  |
| Support from gold traders |  |  |  |  |  |
| usefulness of existing laws |  |  |  |  |  |
| Ways policies are being implemented |  |  |  |  |  |
| Other |  |  |  |  |  |

**Impacts of ASGM’s rise on the 5 capitals in SLA.**

49. How would you estimate the overall impact of ASGM on the way your household make a living? (a lot less = 1; to a lot more = 5)

A lot less A little less No change A little more A lot more

**financial capital**

50. Overall, what level of impact has ASGM activities had on availability of markets/economic activities to your household?

A lot less A little less No change A little more A lot more

~~51. Which of the following activities in your household do you think are linked to ASGM’s rise and how strongly? 1 = not at all, 5 = very strong (Please select one option per line).~~

|  | Not at all | very strong | somewhat strong | strong | very strong |
| --- | --- | --- | --- | --- | --- |
| Cash crop cultivation |  |  |  |  |  |
| Arable crop cultivation |  |  |  |  |  |
| Livestock |  |  |  |  |  |
| Forest resource exploitation |  |  |  |  |  |
| Other mining |  |  |  |  |  |
| Professional |  |  |  |  |  |
| Waged labour |  |  |  |  |  |
| Industry |  |  |  |  |  |
| Agricultural business |  |  |  |  |  |
| Service/shops |  |  |  |  |  |
| Migration/remittances |  |  |  |  |  |
| Other______ |  |  |  |  |  |

**human capital**

52. If applicable, to what extent have ASGM’s revenue enabled you to support education needs of your household? (not at all = 1; very high = 5) Select all that apply.

Not applicable Low Moderate High Very high

primary education

secondary education

tertiary education

53. Have your household members acquired any new skills or knowledge as a result of the surge in small-scale gold mining activities?

1. Yes 2. No

54. To what extent have ASGM’s revenue enabled you to cater for the health needs of your household? (not at all = 1; very high = 5)

1. Not applicable 2. Low 3. Moderate 4. High 5. Very high

55. Which ways have ASGM activities affected the health conditions of your household?

Select all that apply.

1. headaches

2. fever

3. malaria

4. respiratory diseases/coughs

5. skin rashes

6. kidney failure

7. other _________

8. none

**physical capital**

56. Which of the following assets have you acquired as a result of significant contribution from ASGM income (more than 50% of the cost was covered by ASGM income)? Select all that apply.

1. TV

2. Radio

3. Mobile phone

4. shop

5. Car/truck/van

6. Motorbike/motor tricycle

7. Bicycle

8. Motor boat

9. Manual boat

10. House

11. Land for building

12.farmland

13. Other

**social capital**

57. How many local associations, networks and affiliations do your household members belong to in the community?

1. None 2. 1-2 3. 3-5 4. 5-7 5. 8 and above

58. To what extent has the rise of ASGM impacted your participation in any of the following associations or networks? A lot less=1, a lot more=5

|  | A lot less | A little less | No change | A little more | A lot more |
| --- | --- | --- | --- | --- | --- |
| Farmer association |  |  |  |  |  |
| Local mining association |  |  |  |  |  |
| Community watch dog |  |  |  |  |  |
| Local traditional council |  |  |  |  |  |
| Traders/artisans association |  |  |  |  |  |
| Faith based organisation |  |  |  |  |  |
| Community social support group |  |  |  |  |  |
| Community-based NGOs |  |  |  |  |  |
| Local Government committees |  |  |  |  |  |
| Other (specify) |  |  |  |  |  |

**Opportunities, barriers and limits faced by households**

59. What kind(s) of non-farm livelihood activities have you begun or plan to start as a result of ASGM surge in your community?

1. Transport

2. Food vending

3. Trading – selling of physical items

4. Finance/lending

5. Communication/mobile phone services

6. Artisanship – mason, woodwork & carving, welding, steel bender,

7. Real estate/renting houses

8. Other___

9. none

60. How confident are you that your household are able to keep ASGM miners away from mining your farmland? 1= not at all; 5 = very confident.

Not applicable Not confident Somewhat confident Confident Very confident

61. If you have been or you were to be affected by ASGM e.g., through farm invasion, how likely are you to get support through the following association and networks? 1 = not at all; 5= very high.

|  | Not at all | Low | Medium | High | Very high |
| --- | --- | --- | --- | --- | --- |
| Family and friends |  |  |  |  |  |
| Farmer association |  |  |  |  |  |
| Local mining association |  |  |  |  |  |
| Community watch dog |  |  |  |  |  |
| Local traders/artisans/service providers association |  |  |  |  |  |
| Faith based organisation |  |  |  |  |  |
| Community social support association |  |  |  |  |  |
| Community-based NGOs |  |  |  |  |  |
| Local traditional council |  |  |  |  |  |
| Local district assembly |  |  |  |  |  |
| Other Government agencies |  |  |  |  |  |
| International NGOs |  |  |  |  |  |
| other |  |  |  |  |  |

62. For those undertaking mining activities only: how willing are you, under the following circumstances, to undertake measures to ensure responsible mining (Select more than one if applicable) (not at all = 1; very willing = 5)

|  | Not at all | Uncertain | Somewhat willing | Willing | Very willing |
| --- | --- | --- | --- | --- | --- |
| If my colleagues were  doing the same |  |  |  |  |  |
| If there was a subsidy or incentive (e.g. from the local, & central  Government, etc.) |  |  |  |  |  |
| If legal requirements were being enforced |  |  |  |  |  |
| if existing legal requirements were  modified to suit our circumstances |  |  |  |  |  |
| If my household was to be badly impacted by ASGM in the future |  |  |  |  |  |
| If it would reduce the interest rates of the credits I take |  |  |  |  |  |

63. What changes will you like to see regarding the ASGM activities in your community?

1. No change/do nothing

2. stopped outright

3. progressively replaced with

alternative livelihoods

4. Kept but modified to co-exist

with other livelihood options

5. other

64. Are you willing to participate in future aspects of the research and provide your contact details?

1. Yes 2. No

====================================END=========================================
