# Supplementary material for: Joined-up governance for more complementary interactions between expanding artisanal small-scale gold mining and agriculture: Insights from Ghana
Source: PLoS One. 2024 Apr 4;19(4):e0298392. doi: 10.1371/journal.pone.0298392 (PMC10994392; doi:10.1371/journal.pone.0298392)
Supplement: S2 File — (DOCX) [file pone.0298392.s003.docx]

**Focus group discussions (FGDs)**

Questions:

1. How do ASGM and agriculture interact at the moment?

2. What circumstances affects these interactions, and which is most important?

3. Group exercise for participants.

Using post it notes, please submit your ideas on how ASGM has affected the following areas in the community since 2010:

| sectors | effects |
| --- | --- |
| Environment |  |
| Physical assets like roads and water |  |
| Jobs and finances |  |
| Health and education |  |
| Population composition and quantity |  |
| Family, organizational membership, and social networks |  |

*(NB: A bigger version of the table above will be developed, and participants asked to put their ideas on post it notes and stick to each section, and discussion held afterwards.)*

9. What actions have farmers undertaken when farmlands are invaded by miners?

10. What local measures, if any, have farmers adopted to reclaim/restore degraded lands?

11. What shifts, if any, have taken place in farming methods to adapt to ASGM insurgence?

12. Which groups have offered support in dealing with undesirable ASGM impacts?

13. What kinds of reforms could help co-existence of ASGM and agriculture be more sustainable?
