# Supplementary material for: Joined-up governance for more complementary interactions between expanding artisanal small-scale gold mining and agriculture: Insights from Ghana
Source: PLoS One. 2024 Apr 4;19(4):e0298392. doi: 10.1371/journal.pone.0298392 (PMC10994392; doi:10.1371/journal.pone.0298392)
Supplement: S3 File — (DOCX) [file pone.0298392.s004.docx]

**Oral history interview**

Introduction:

This interview seeks to explore main livelihood activities that you undertook, from the past to the present and to find out any changes that have occurred and the factors that led to those changes.

Therefore, please note the format of the interview is such that I will first ask questions about the 1990s, then the mid 2000s and finally the present.

**Interview Questions:**

1. Please describe your main livelihood activities during the early to mid-1990s (*before ASGM boom in the community*) and why you chose those activities?

2. Can you tell me about any credits/loans/subsidies, if any, that you accessed around that time?

3. Can you tell me about the availability and quality of labour for hire around that time?

4. Can you please tell me about the environment, its quality and how it influenced your livelihoods in the mid 1990s?

5. Can you tell me about the state of the roads and physical infrastructure in the mid 1990s?

6. Tell me about your social networks and linkages in the 1990s– who did you depend on when the going got tough?

7. How have your livelihood activities changed from the mid 1990s until the mid 2000s and why?

8. How have livelihood activities changed since 2010 (*after ASGM boom in the community*) and what were the main reasons for these changes?

9. How has the rise of mining impacted your current livelihood activities (the advantages and disadvantages for you)?

10. Can you see any new and emerging livelihood options in your community?

11. What would you require to harness those opportunities?

12. What barriers and limitations hinder you from achieving your desired livelihood outcomes?

**NB**

*Personal details on interviewees will be compiled from the questionnaire surveys that they would have previously completed.*
