# Supplementary material for: Joined-up governance for more complementary interactions between expanding artisanal small-scale gold mining and agriculture: Insights from Ghana
Source: PLoS One. 2024 Apr 4;19(4):e0298392. doi: 10.1371/journal.pone.0298392 (PMC10994392; doi:10.1371/journal.pone.0298392)
Supplement: S4 File — (DOCX) [file pone.0298392.s005.docx]

**Environmental inventory checklist (for mine sites on farmland)**

| **A. FUNDAMENTALS** | **Notes** | |
| --- | --- | --- |
| Date |  | |
| Village |  | |
| Type and size of farmland |  | |
| Type of ASGM |  | |
| GPS coordinates for ASGM site |  | |
| estimated age of ASGM site |  | |
| Topography/slope |  | |
|  |  | |
| **SAMPLING** |  | |
| Size of mined area (length * breadth) |  | |
| Number of shafts/adits (n) (for underground); average circumference =(∑π*diameter)/n |  | |
| Size of pit/volume of ponds (for surface mines) = πr^2^h (estimate diameter visually; depth of pit/pond – measure using dip stick, where possible or estimate visually) |  | |
| Nature of route to mine site |  | |
|  | | |
| **B. GROUND CONDITION AND SITE FEATURES** |  |  |
| Machinery & equipment used | Quantity | Description |
|  |  |  |
|  |  |  |
|  |  |  |
|  |  |  |
|  |  |  |
|  |  |  |
|  |  |  |
|  |  |  |
|  |  |  |
|  |  |  |
|  |  |  |
|  |  |  |
|  |  |  |
|  |  |  |
|  |  |  |
|  |  |  |
|  |  |  |
|  |  |  |
|  |  |  |
|  |  |  |
| Any chemicals or reagents used for operation  (e.g., mercury, cyanide, acid etc.) | Estimated consumption | Description |
|  |  |  |
|  |  |  |
|  |  |  |
|  |  |  |
|  |  |  |
|  |  |  |
|  |  |  |
|  |  |  |
|  |  |  |
|  |  |  |
| Water use & disposal pattern during processing |  |  |
| Source of water (e.g., river, stream, bore hole, pond, etc.) | Means of wastewater discharge | Discharge points (e.g., into river, stream, wetland, farm etc). |
|  |  |  |
|  |  |  |
|  |  |  |
|  |  |  |
|  |  |  |
|  |  |  |
|  |  |  |
|  |  |  |
|  |  |  |
|  |  |  |
|  |  |  |
|  |  |  |
|  |  |  |
|  |  |  |
|  |  |  |
|  |  |  |
|  |  |  |
|  |  |  |

**Transect walk - farmland degradation assessment (environmental visual assessment)**

**1. Farmland areas impacted by ASGM**

| **Degradation types** | **Description** |
| --- | --- |
| **Physical degradation** |  |
| Extent of bare ground, deforested spaces, health status of vegetation - healthy/stunted growth/sparse? |  |
| Crusting, sealing, compaction/inhibited aeration, subsidence/terrain deformation, others? |  |
| Number and sizes of mine waste – heaps of soil, excavated pits, ponds, waterlog, litter etc.?  condition of pits, heaps stable? |  |
| water erosion, runoff/ loss of topsoil;  wind erosion/ overblowing, |  |
| **Chemical degradation** |  |
| Signs of soil contaminated by mercury, cyanide, diesel, kerosene, petrol, grease, gasoil, etc? |  |
| Signs of acidification, nutrient losses (erosion), e.g., stunted grass growth/wilting grass?  Signs of salinization/alkalinization, leaching? |  |
| Contaminated water/ponds – flowing/stagnant, silted, turbid, oil-film on surface, water colour, signs of dead fishes and other animals, other.  status of water for irrigation purposes? |  |
| **Biological degradation** |  |
| Loss of biodiversity, decline in soil organic matter, soil-borne pathogens, emission of greenhouse gases, loss of soil carbon sink capacity, loss of soil structure |  |
| **Nature of reclamation or restoration** |  |
| Is it vegetation, soil, water, habitat etc?  Method used – earthworks machinery, local farm implements, natural regeneration?  revegetation full or partial? |  |

**2. Farmland areas not impacted by ASGM**

*(Please tick alongside the ticked spots only, for any indicator that is present)*

| Visual Indicator | Types of Soil and Land degradation | | | | | |
| --- | --- | --- | --- | --- | --- | --- |
|  | Water Erosion | Wind  Erosion | Salinity or Alkalinity | Chemical degradation | Physical degradation | Biological degradation |
| Rills | √ | × | × | × | × | × |
| Gullies | √ | × | × | × | × | × |
| Pedestals | √ | √ | × | × | × | × |
| Armour layer | √ | √ | × | × | × | × |
| Accumulations of soil around clumps of vegetation or upslope of trees, fences or other barriers | √ | √ | × | × | × | × |
| Deposits of soil on gentle slopes | √ | × | × | × | × | × |
| Exposed roots or parent material | √ | √ | × | × | × | × |
| Muddy water/mudflows during and shortly after storms | √ | × | × | × | × | × |
| Sedimentation in streams and reservoirs | √ | × | × | × | × | × |
| Dust storms/clouds | × | √ | × | × | × | × |
| Sandy layer on soil surface | × | √ | × | × | × | × |
| Parallel furrows in clay soil or  ripples in sandy soil | × | √ | × | × | × | × |
| Bare or barren spots | √ | √ | √ | √ | × | × |
| Efflorescence | × | × | √ | × | × | × |
| Soil particles unstable in water | × | × | √ | × | × | √ |
| Nutrient deficiency/toxicity  symptoms evident on plants | √ | × | × | √ | × | √ |
| Increased incidence of plant  disease/morphological  irregularities (e.g. stunting) | × | × | √ | √ | √ | × |
| Decreasing yields | √ | √ | √ | √ | √ | √ |
| Changes in vegetation species | √ | × | √ | √ | × | × |
| Plough pan | × | × | × | × | √ | × |
| Restricted rooting depth | √ | × | × | × | √ | × |
| Structural degradation, including compaction | × | × | √ | × | √ | × |
| Poor response to fertilizers | x | × | × | √ | × | √ |
| Decrease in organic matter  (lighter-coloured soils) | √ | × | √ | × | × | √ |
| Increased sealing, crusting and  run-off; reduced soil water | × | × | √ | √ | √ | √ |
| Decrease in number of  earthworms/ants and similar | × | × | × | × | × | √ |

*Source: Adapted from Stocking, M. & Murnaghan, N. (2001) Handbook for the field assessment of land degradation.*

3. Any further information regarding general ground condition and features on the farmland

|  |
| --- |
