# Supplementary material for: Joined-up governance for more complementary interactions between expanding artisanal small-scale gold mining and agriculture: Insights from Ghana
Source: PLoS One. 2024 Apr 4;19(4):e0298392. doi: 10.1371/journal.pone.0298392 (PMC10994392; doi:10.1371/journal.pone.0298392)
Supplement: S5 File — (DOCX) [file pone.0298392.s006.docx]

**Sources of public datasets used in the study.**

Mining Acts:

Minerals and Mining Law, 2006 (Act 703)

Minerals Commission Act, 1993 (Act 450)

Minerals and Mining (Amendment) Act, 2015 (Act 900)

Minerals and Mining (Amendment) Act, 2019 (Act 995)

Source: <https://www.mincom.gov.gh/acts/>

------------------------------------------------------------------------------------------------------------------------------

Regulations:

Ghana Government. MINERALS AND MINING (COMPENSATION AND RESETTLEMENT) 2012 (LI 2175).

Ghana Government. MINERALS AND MINING (HEALTH, SAFETY) 2012 (LI 2182).

Source: https://www.mincom.gov.gh/regulations/

Ghana Government. ENVIRONMENTAL ASSESSMENT REGULATIONS, 1999 (LI 1652).

Source: <http://www.epa.gov.gh/epa/regulations>

<https://www.fao.org/faolex/results/details/en/c/LEX-FAOC078169/>

-------------------------------------------------------------------------------------------------------------------------------------

Policy:

Ghana Government (Minerals Commission). MINERALS AND MINING POLICY OF GHANA, 2014.

Source: <https://www.mincom.gov.gh/wp-content/uploads/2021/06/Mineral-and-Mining-Policy-Ghana.pdf>

Ghana Government (Minerals Commission). Small scale and Community Mining - Operational Manual, 2021. 2021.

<https://www.mincom.gov.gh/wp-content/uploads/2021/11/Small-Scale-and-Community-Mining-Operational-Manual-Sep.-2021-1.pdf>

Ghana Government: Min of Water Resources W and H. Riparian Buffer Zone Policy for Managing Freshwater Bodies in Ghana. 2013.

Source: <https://www.wrc-gh.org/dmsdocument/93>

National Agriculture investment Plan (IFJ) – Ghana (2018-2021)

<https://mofa.gov.gh/site/publications/policies-plans>

--------------------------------------------------------------------------------------------------------------------------------------

Public sector reports:

Ghana Audit Service. Performance audit report of the Auditor-General on regulating reclamation activities at small-scale mining sites. 2021;

Source: <https://audit.gov.gh/files/audit_reports/Performance_Audit_Report_of_the_Auditor-General_on_Regulating_Reclamation_Activities_at_Small-Scale_Mining_Sites.pdf>

Atiwa West District. Composite Budget for 2021-2024: Programme Based Budget estimates for 2021. 2021.

Source: <https://mofep.gov.gh/sites/default/files/composite-budget/2021/ER/Atiwa_West.pdf>

Geological Survey of Denmark and Greenland. Artisanal and Small scale Mining Handbook for Ghana with a regional perspective [Internet]. 2017. 160 p.

Available from: <http://www.oagsafrica.org/images/training/WP3SUCCESSSTORY/FINAL-Artisanal-Handbook-for-Ghana.pdf>
